# A complex of MAST1 and 14-3-3η regulates Tau phosphorylation in the developing cortex

**DOI:** 10.1073/pnas.2617534123

**Published:** 2026-09-17

**Authors:** Sumire Antonioli, Patrick Heisterkamp, Weiqiang Chen, Dorothea Anrather, Markus Hartl, Maria Fernanda Martinez-Reza, Ratna Tripathy, Michael Schutzbier, Karl Mechtler, David A. Keays, Thomas A. Leonard

**Affiliations:** ^a^ Max Perutz Labs, Medical University of Vienna, Vienna 1030, Austria; ^b^ Vienna BioCenter PhD Program, A Doctoral School of the University of Vienna and the Medical University of Vienna, Vienna A-1030, Austria; ^c^Division of Neurobiology, Department of Biology, Ludwig-Maximilians-University Munich, Planegg-Martinsried 82152, Germany; ^d^ Max Perutz Labs, Mass Spectrometry Facility, Vienna 1030, Austria; ^e^ Department of Biochemistry and Cell Biology, Center for Molecular Biology, University of Vienna, Vienna 1030, Austria; ^f^Division of Physiological Genomics, Biomedical Center, Ludwig-Maximilians-University Munich, Planegg-Martinsried 82152, Germany; ^g^Institute of Molecular Pathology, Vienna 1030, Austria

**Keywords:** serine/threonine kinase, phosphorylation, microtubules, corpus callosum, neurodevelopment

## Abstract

The MAST family of serine/threonine kinases has been implicated in a spectrum of human neurodevelopmental disorders. However, little is known about their biological function or regulation. Seeking to fill these gaps in our knowledge, we have identified upstream and downstream partners of MAST1. 14-3-3η, a neuronal 14-3-3 paralog, specifically interacts with MAST1 at two regulatory serines, S90 and S161. p21-activated kinase (PAK), a neuronal regulator of the actin cytoskeleton, phosphorylates MAST1 to regulate its interaction with 14-3-3η. Exploiting mouse models of human Mega-Corpus-Callosum Syndrome (MCC) and whole brain phosphoproteomics, we identify the microtubule-associated protein Tau as a candidate substrate of MAST1. We show that pathogenic MAST1 mutations perturb protein function either through misfolding or attenuation of kinase activity. Our data are consistent with a model in which the MAST kinases couple PAK, a neuronal regulator of the actin cytoskeleton, to microtubule remodeling during the differentiation and specification of cortical neurons.

## Significance

Mutations in the microtubule-associated serine/threonine (MAST) kinases are increasingly being associated with neurodevelopment disorders, yet little is known about their structure, substrate specificity, or role in neurodevelopment. We reveal several interaction partners of MAST1, including upstream regulators and downstream substrates, recapitulate morphological changes in brain structure in a mouse model of a patient-derived MAST1 mutation associated with Mega-Corpus-Callosum Syndrome (MCC), and determine the molecular consequences of disease-associated mutations on MAST1 activity in vitro. Our work suggests that the MAST kinases may be intermediary signal transducers that link the actin and microtubule cytoskeletons, setting the stage for further investigation into their role(s) in neurodevelopment.

The differentiation of cortical neurons is an essential and tightly regulated process, dysregulation of which is associated with a variety of neurodevelopmental disorders (NDDs). A rare NDD called Mega-Corpus-Callosum Syndrome with Cerebellar Hypoplasia and Cortical Malformations (MCC-CH-CM) is characterized by enlargement of the corpus callosum, epilepsy, and severe cognitive and motor impairment (1). Whole exome sequencing of a cohort of seven patients revealed six harbored de novo mutations in a gene called Microtubule-Associated Serine/Threonine Kinase 1 (MAST1). A mouse model that recapitulates a heterozygous trinucleotide deletion (L278del) of MAST1 identified in an MCC-CH-CM patient mirrors multiple aspects of the disease, including the enlarged corpus callosum. The identification of additional MCC patients bearing mutations in MAST1, as well as de novo mutations in MAST1, MAST3, and MAST4 associated with epilepsy, autism, cerebral palsy, and intellectual disability (2–9) has provided further evidence for the involvement of the MAST kinases in neurodevelopment. Despite their association with human NDDs, very little is known about the biological function, structure, or regulation of any MAST kinase.

The MAST kinases are large, ~200 kDa proteins that first arose ~600 Mya in Cnidaria and Ctenophora, the first organisms with a primitive nervous system (10). They contain three domains; an N-terminal domain of unknown function (DUF) which harbors a four-helix bundle (4HB), a catalytic Ser/Thr kinase domain, and a C-terminal PDZ domain. The kinase domain belongs to the AGC family of protein kinases, characterized by the presence of a regulatory C-terminal tail (11). Although many eukaryotic protein kinases are regulated by activation loop phosphorylation (12), the MAST kinases possess a glutamine in place of the canonical serine or threonine.

MAST1 is expressed in postmitotic cortical neurons throughout neurodevelopment (1, 13, 14), associating with the microtubule cytoskeleton via unknown microtubule associated proteins (MAPs) (1). It has been reported that the PDZ domain of MAST1 interacts with the adaptor protein β2-syntrophin, bridging it to the dystrophin/utrophin network at postsynaptic densities (13). Little is known about MAST substrates. MAST1/2/3 and KIN-4 (the sole homolog of MAST kinases in *Caenorhabditis elegans*) reportedly phosphorylate the C-terminus of PTEN in a PDZ domain-dependent manner, however, this interaction has not been confirmed in vivo (15–17). In addition, Mitogen-Activated Protein Kinase Kinase (MEK) (18) and cAMP-regulated phosphoprotein of 16 kDa (ARPP-16) (19) are also reported substrates. However, these findings are largely based on immunoprecipitated proteins and lack kinase-dead controls, while the substrates neither conform to canonical AGC kinase substrate motifs, nor establish a functional link to development of the corpus callosum.

Here, we identify 14-3-3η as an interaction partner of MAST1. We show that 14-3-3η binds specifically to two conserved phosphorylation sites in MAST1, S90 and S161. We identify p21-activated kinase (PAK) as the upstream kinase that phosphorylates MAST1 on S90, and S161 as a putative regulatory autophosphorylation site. We demonstrate that MAST1 phosphorylated on either S90 or S161 forms a stable complex with 14-3-3η in vitro. Using two mouse models of patient-derived MCC-CH-CM mutations, we identify the microtubule-associated protein Tau as a candidate substrate of MAST1 and demonstrate that MAST1 phosphorylates Tau in vitro. Finally, we show that MCC-CH-CM-associated disease mutations perturb MAST1 kinase activity either through misfolding of its 4HB domain or disruption of its catalytic site.

## Results

### MAST1 Interacts with 14-3-3 Proteins via S90 and S161.

**Fig. 1.**
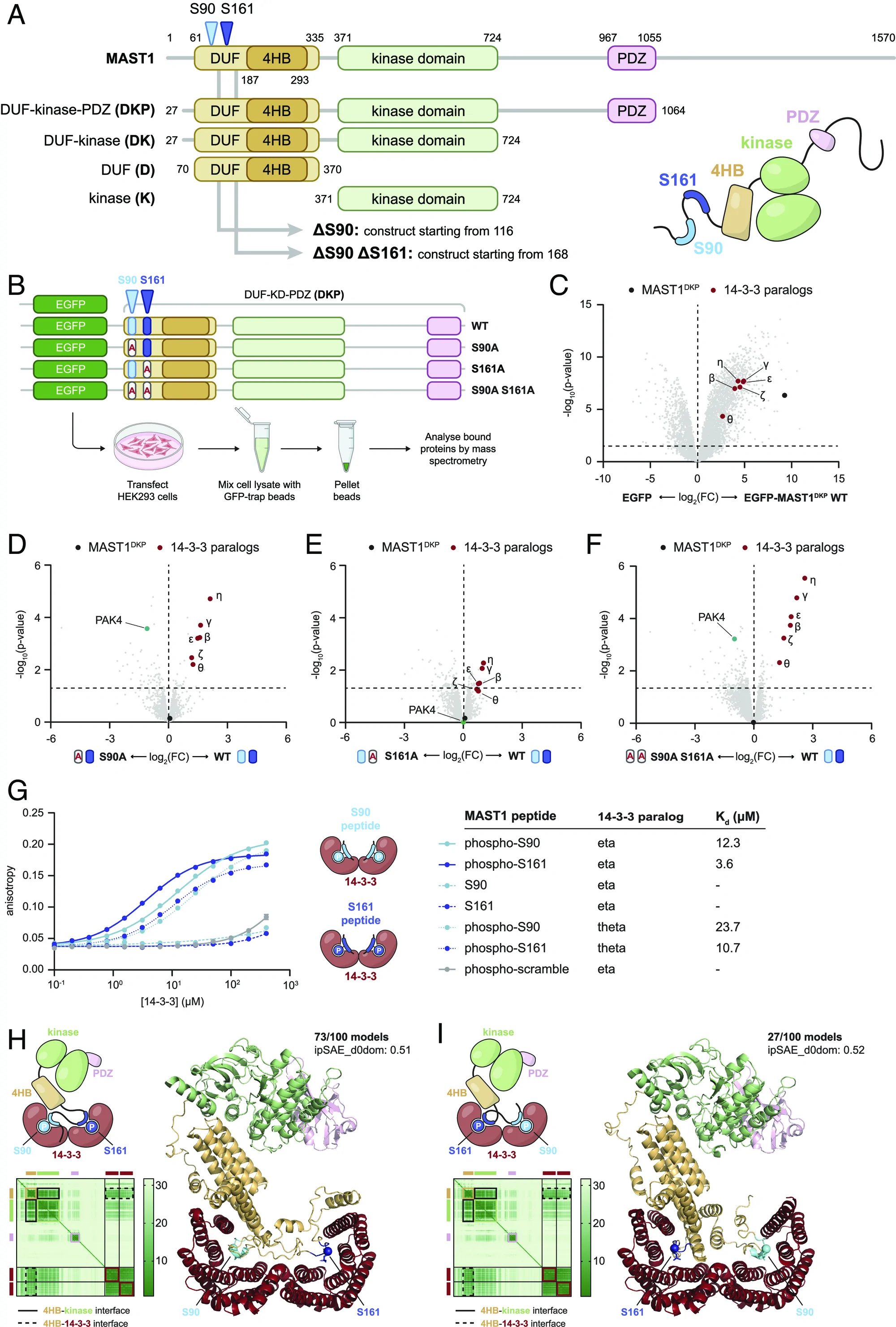
MAST1 interacts with 14-3-3 proteins via S90 and S161. (*A*) Domain architecture of *Mus musculus* MAST1. Construct boundaries and nomenclature for all constructs used in this study are defined. DUF = domain of unknown function, 4HB = four-helix bundle, PDZ = postsynaptic density protein (PDS95) *Drosophila* disc large tumor suppressor (DlgA) and Zonula occludens-1 protein (zo-1). *Right*: schematic representation of MAST1 to be employed throughout the figures. (*B*) Constructs and experimental setup for EGFP-MAST1^DKP^ AP-MS in HEK293 cells. (*C*) Volcano plot displaying the enriched interactome of EGFP-MAST1^DKP^ (*Right*) vs. EGFP (*Left*) from AP-MS, *n* = 3. The horizontal dashed line represents significance cutoff (*P* < 0.05). (*D*) Volcano plot displaying the enriched interactome of EGFP-MAST1^DKP^ WT (*Right*) vs. EGFP-MAST1^DKP^ S90A (*Left*) from AP-MS, *n* = 3. The horizontal dashed line represents significance cutoff (*P* < 0.05). (*E*) Volcano plot displaying the enriched interactome of EGFP-MAST1^DKP^ WT (*Right*) vs. EGFP-MAST1^DKP^ S161A (*Left*) from AP-MS, *n* = 3. The horizontal dashed line represents significance cutoff (*P* < 0.05). (*F*) Volcano plot displaying the enriched interactome of EGFP-MAST1^DKP^ WT (*Right*) vs. EGFP-MAST1^DKP^ S90A S161A (*Left*) from AP-MS, *n* = 3. The horizontal dashed line represents significance cutoff (*P* < 0.05). (*G*) Binding curves for 14-3-3η or θ and MAST1 peptides spanning S90 or S161, measured by fluorescence anisotropy. Data are presented as mean values ± SE, *n* = 3. Data were fit with a one-site binding model and the K_d_ derived from curve fitting. (*H*) AlphaFold 3 (27) model and PAE plot of 14-3-3η dimer (red) and MAST1 FL showing DUF (yellow), kinase domain (green), and PDZ domain (pink), with phosphoserine modification for S90 (light blue sphere) and S161 (dark blue sphere). One representative model of 73 where S90 binds on the side of the DUF. (*I*) AlphaFold 3 (27) model and PAE plot of 14-3-3η dimer (red) and MAST1 FL showing DUF (yellow), kinase domain (green), and PDZ domain (pink), with phosphoserine modification for S90 (light blue sphere) and S161 (dark blue sphere). One representative model of 27 where S161 binds on the side of the DUF.

To shed light on the function and regulation of the MAST kinases, we first sought to identify MAST1 interactors by affinity-purification mass spectrometry (AP-MS) in HEK293 cells. All MAST1 constructs employed in this study are depicted in Fig. 1*A*. MAST1^DKP^ WT, which includes all three folded domains, was N-terminally tagged with EGFP and overexpressed in HEK293 cells (Fig. 1*B*). Comparison of the EGFP-MAST1-bound proteins with an EGFP-only control revealed a MAST1-dependent 6- to 30-fold enrichment of six 14-3-3 paralogs (η, γ, ε, β, ζ, and θ) (Fig. 1*C* and Datasets S1 and S2) (20). 14-3-3 proteins are constitutively dimeric and are known to bind specifically to phosphorylated serines (21). Analysis of MAST1’s amino acid sequence by 14-3-3Pred (22) revealed two putative 14-3-3 binding motifs (23, 24) surrounding S90 and S161. Both sites exhibit near-invariant sequence conservation over 600 My and phosphorylation of each site has been observed experimentally in human cells (25, 26).

To determine whether phosphorylated S90 and S161 interact with 14-3-3, we performed affinity purification mass spectrometry (AP-MS) with MAST1^DKP^ WT and S90A or S161A point mutants (Fig. 1*B*) and made pairwise comparisons of their interactomes. Mutation of either S90 or S161 resulted in a significant loss of 14-3-3 binding to MAST1 (Fig. 1 *D* and *E*), while double mutation of both residues resulted in an additional loss of 14-3-3 proteins (Fig. 1*F*). Comparison of single-mutant to double-mutant samples revealed that a single phosphosite is sufficient to enrich 14-3-3, with S90 contributing a stronger interaction (*SI Appendix*, Fig. S1 *A* and *B*). EGFP-MAST1^DKP^ immobilized on GFP-trap beads and incubated with the lysate of day 120 dorsal brain organoids similarly enriched all 14-3-3 paralogs when compared to the S90A S161A double-mutant (*SI Appendix*, Fig. S1*C* and Dataset S3) (28).

Truncation constructs of MAST1 lacking S90, or both S90 and S161 (*SI Appendix*, Fig. S1*D*) provide further evidence of 14-3-3 binding. We observed significant enrichment of the 14-3-3 paralogs by MAST1^DKP^ when compared to either MAST1^DKP^ ΔS90 (*SI Appendix*, Fig. S1*E*) or MAST1^DKP^ ΔS90 ΔS161 (*SI Appendix*, Fig. S1*F*). The absence of 14-3-3 enrichment in MAST1^DKP^ ΔS90, which includes S161 but not S90 (*SI Appendix*, Fig. S1*G*), suggests that the N-terminal sequence that includes S90 may contribute additional interactions beyond the phosphorylation motif. 14-3-3η was reproducibly the most highly enriched paralog of the six, despite having the lowest abundance in HEK293 cells (29) (*SI Appendix*, Fig. S1*H*). Consistently, 14-3-3η was the most significantly enriched paralog from dorsal brain organoids (*SI Appendix*, Fig. S1*C*). Expression of 14-3-3η is highly enriched in the brain and is ubiquitous in all brain structures (30–33), while single-cell sequencing has shown that MAST1 and 14-3-3η are coexpressed with the callosal projection neuron marker SATB2 in developing cortical neurons of mouse embryos (34) (*SI Appendix*, Fig. S2 *A*–*D*).

We next confirmed the interaction between 14-3-3η and MAST1 by determining its binding affinity to phosphorylated peptides corresponding to the S90 and S161 motifs in vitro (Fig. 1*G*). We confirmed the correct mass of all recombinant proteins and derivatives thereof employed in this study by mass spectrometry (*SI Appendix*, Figs. S3 and S4). Consistent with the AP-MS results, 14-3-3η bound to both phospho-S90 (p-S90) and phospho-S161 (p-S161) peptides, but neither to their unphosphorylated counterparts nor a phosphopeptide with a scrambled amino acid sequence. 14-3-3θ, which exhibited the lowest enrichment by AP-MS, bound MAST1 phosphopeptides with a correspondingly lower affinity. In summary, 14-3-3η binds to MAST1 phosphorylated on S90 and S161.

To visualize a potential interaction between MAST1 and 14-3-3η, we predicted the structure of full-length MAST1 and 14-3-3η, using AlphaFold 3 (27). Accurate structure inference by AlphaFold depends on the detection of evolutionary covariance of amino acids, for which the accuracy of the input multiple sequence alignment (MSA) is crucial (35). Since AlphaFold 3 generated an MSA for MAST1 that contained just 12% of bona fide MAST sequences (*SI Appendix*, Fig. S5*A*), we employed a custom pipeline previously developed (36) to generate a deep and taxonomically balanced MSA containing exclusively MAST sequences for structure prediction. We predicted the structure of MAST1 phosphorylated on S90 and S161 in complex with dimeric 14-3-3η with either 1:2 or 2:2 stoichiometry based on known stoichiometries of 14-3-3 proteins in complex with protein kinases (37–40). We ran the structure inference step with 100 independent seeds and evaluated model convergence and predicted error estimates. For a 1:2 MAST1:14-3-3 complex, AlphaFold converged on a singular arrangement of the 4HB and kinase domains of MAST1 and the two chains of 14-3-3η (Fig. 1 *H* and *I* and *SI Appendix*, Fig. S5*B*), into which AlphaFold placed p-S90 and p-S161 in alternative conformations. 73/100 models placed p-S90 in the 14-3-3 protomer nearest to the 4HB domain (Fig. 1*H*), while 27/100 models placed p-S90 in the other protomer (Fig. 1*I*). Superposition of the resulting structure with an experimentally determined structure of 14-3-3η in complex with a bound phospho-peptide (PDB ID 2C63) (41) revealed that both S90 and S161 of MAST1 were consistently placed in the same position as the bound phospho-serine of the ligand (*SI Appendix*, Fig. S5*C*).

To assess the convergence of the 100 models, we measured the Euclidean distance between two pairs of residues lining each domain interface (*SI Appendix*, Fig. S5*D*). Euclidean distance plots corresponding to p-S90:14-3-3 (*SI Appendix*, Fig. S5*E*), p-S161:14-3-3 (*SI Appendix*, Fig. S5*F*), 4HB:14-3-3 (*SI Appendix*, Fig. S5*G*), and 4HB:kinase domain (*SI Appendix*, Fig. S5*H*) interfaces indicate 100% model convergence at the level of domain arrangement, with 73/100 models placing p-S90 in the 14-3-3 protomer closest to MAST1 and 27/100 models inverting the placement of p-S90 and p-S161. The PDZ domain of MAST1 did not exhibit model convergence (*SI Appendix*, Fig. S5*I*). Superposition of a second MAST1 chain in the same arrangement results in steric clashes between the kinase domains of the two protomers (*SI Appendix*, Fig. S5*J*), indicating a likely stoichiometry of 1:2 MAST1:14-3-3. Consistent with this, AlphaFold predictions of a 2:2 complex failed to exhibit any model convergence.

In summary, MAST1 binds to 14-3-3 proteins in cells, in vitro and in silico.

### PAK4 Phosphorylates MAST1 on S90.

**Fig. 2.**
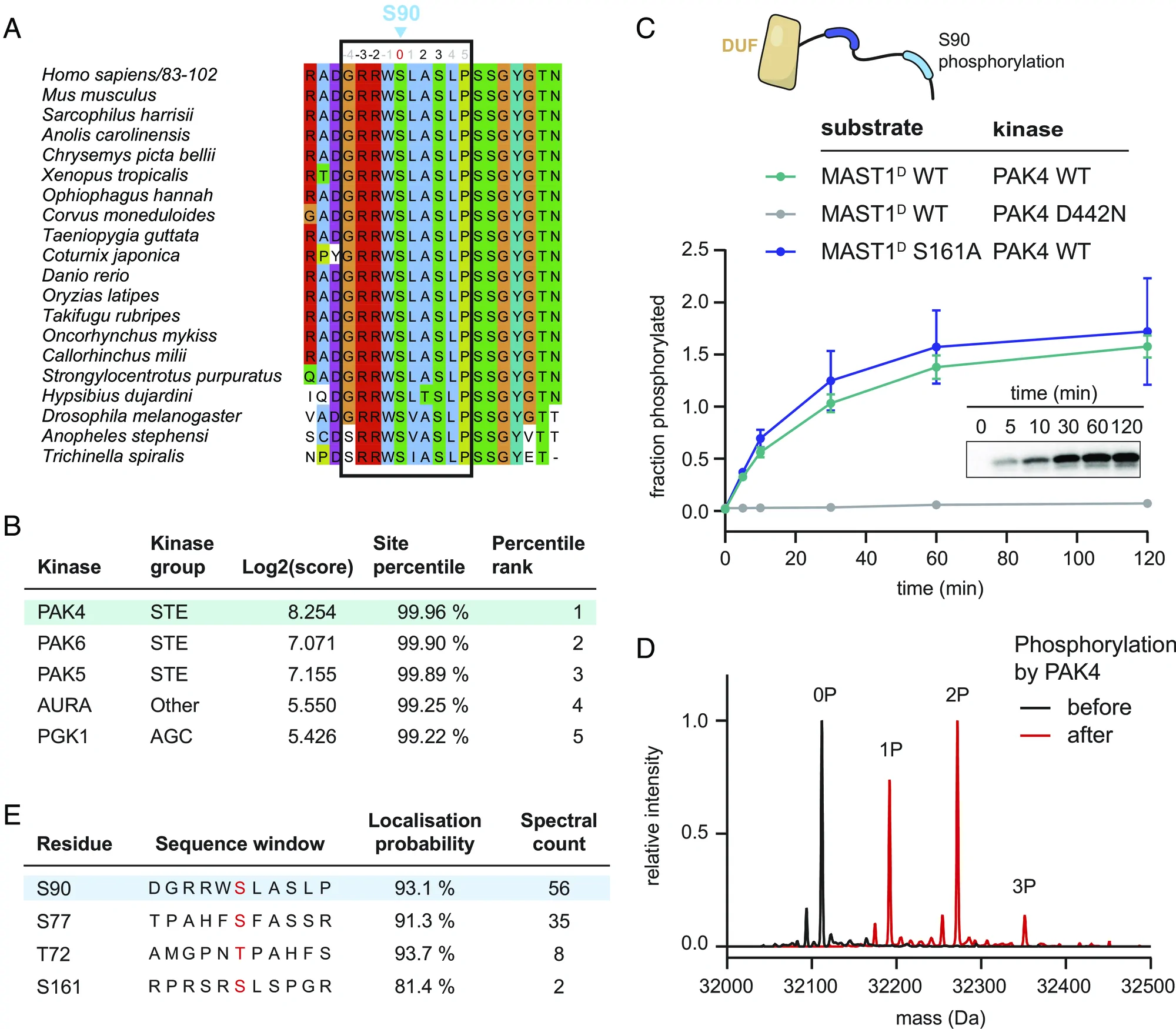
PAK4 phosphorylates MAST1 on S90. (*A*) Multiple sequence alignment of MAST1 residues surrounding S90 from *Nematoda* to *Mammalia*. S90 residue highlighted above, sequence used for ScoreSite prediction outlined in black box. (*B*) ScoreSite (42) prediction results using input sequence GRRWs*LASLP (s* represents S90 phosphosite). Top five hits shown in table, PAK4 highlighted in turquoise. (*C*) Radiometric kinase assay by PAK4 WT or PAK4 kinase dead (D442N) on MAST1^D^ WT or S161A. Data are presented as mean values ± SE, *n* = 3. Representative phosphorimage shown in *Inset*. (*D*) Intact MS analysis of MAST1^D^ before (black) and after (red) phosphorylation by PAK4 WT shown in panel *C*. Intensity of both samples were normalized to a scale of 0 to 1. Addition of phosphate (1P, 2P, etc.) detected by each 80 Da shift. (*E*) Tandem MS analysis of MAST1^D^ after phosphorylation by PAK4 WT shown in panel *C*. Phosphorylated residues are indicated in red. Top three sites (by spectral count) shown in table, as well as S161. S90 highlighted in light blue. See Dataset S3 for full data.

The phosphorylation-dependent interaction of MAST1 with 14-3-3η immediately begs the question of which kinase(s) phosphorylate S90 and S161. To identify candidate upstream kinases for the conserved S90 site (Fig. 2*A*), we employed the ScoreSite prediction algorithm (42), which identified PAK4-6 as the highest ranked kinases (Fig. 2*B*). Curiously, we observed significant enrichment of PAK4 in all AP-MS experiments involving MAST1 S90A (Fig. 1 *D* and *F*), suggesting that endogenous PAK4 may have been trapped on a nonphosphorylatable substrate. While PAK4 is relatively abundant in HEK293 cells (280 nM), PAK5 and PAK6 are not detected (43) (*SI Appendix*, Fig. S6*A*). PAK4-6 are group II PAKs with a consensus motif that differs from group I PAKs (PAK1-3) at positions P+2 and P+3 (*SI Appendix*, Fig. S6*B*). PAK4-6 recognize an alanine at the P+2 position, in contrast to the tyrosine or phospho-tyrosine favored by PAK1-3, and PAK4 has a strong preference for a serine at the P+3 position (44). The S90 motif of MAST1 encodes alanine and serine in the P+2 and P+3 positions respectively (Fig. 2*A*). Consistent with PAK4 being the upstream kinase of MAST1 in HEK293 cells, we observed that purified, recombinant PAK4 phosphorylates MAST1^D^ in an in vitro radiometric kinase assay while a kinase-dead mutant did not (Fig. 2*C*). Mass spectrometry analysis revealed that S90 was phosphorylated with the highest spectral count (Fig. 2 *D* and *E* and Dataset S4) (45). We also observed S77 to be phosphorylated, likely due to the similarity in the surrounding amino acid sequence. S161 phosphorylation was detected with only 2 spectral counts and MAST1^D^ S161A phosphorylation kinetics by PAK4 were unchanged (Fig. 2*C*), indicating that S161 is not a significant target of PAK4.

In summary, PAK4 phosphorylates MAST1 on S90 in vitro and was observed to interact specifically with MAST1 S90A in cells.

### MAST1 S161 Is a Putative Autoregulatory Motif.

Having identified the upstream kinase for S90, we next sought to identify the kinase that phosphorylates S161. In contrast to PAK4, which was specifically enriched by MAST1^DKP^ S90A by AP-MS (Fig. 1 *D* and *F*), we did not observe a significant enrichment of any protein kinase by MAST1^DKP^ S161A (Fig. 1*E*). Inspection of the conserved motif surrounding S161 (*SI Appendix*, Fig. S7*A*) revealed the presence of a canonical AGC kinase consensus sequence, characterized by arginine in the P-5 and P-3 positions, as well as a large hydrophobic residue in the P+1 position (*SI Appendix*, Fig. S7*B*) (11). This motif is near invariant in all MAST kinases (*SI Appendix*, Fig. S7*C*), implying an evolutionarily conserved function. Since MAST kinases are, themselves, members of the AGC family and exhibit conservation of key residues required for substrate binding (*SI Appendix*, Fig. S7*D*), this observation suggested that S161 may represent an autoregulatory phosphorylation site. In an in vitro kinase assay, recombinant MAST1^DK^ ΔS90 (*SI Appendix*, Fig. S7 *E* and *F*) incubated with ATP was able to autophosphorylate, while a kinase-dead MAST1 was completely inactive (*SI Appendix*, Fig. S7 *G* and *H*). To determine the site-specificity of the reaction, we performed tandem mass spectrometry, which revealed phosphorylation of S161 (*SI Appendix*, Fig. S7*I* and Dataset S4) (46). However, the reaction was inefficient and resulted in multiple phosphorylated sites, suggesting that unknown factors may be required for full and specific MAST1 activity. Finally, we measured the binding affinity of MAST1^DK^ ΔS90 ΔS161 (*SI Appendix*, Fig. S7 *J* and *K*) for a peptide corresponding to the S161 motif (*SI Appendix*, Fig. S7*L*). A peptide bearing the native sequence of MAST1 bound to the kinase with a low affinity consistent with the known strength of kinase–substrate interactions (47). Mutation of the arginines at P-3 and P-5 reduced the binding affinity, which was further reduced by mutation of the leucine in the P+1 position (*SI Appendix*, Fig. S7*L*). These findings are consistent with a specific interaction of the conserved S161 motif with the MAST1 kinase domain. In summary, MAST1 can undergo autophosphorylation at S161, but the factors which control the rate and specificity of the reaction are unknown.

### Reconstitution of MAST1:14-3-3η Complexes In Vitro.

**Fig. 3.**
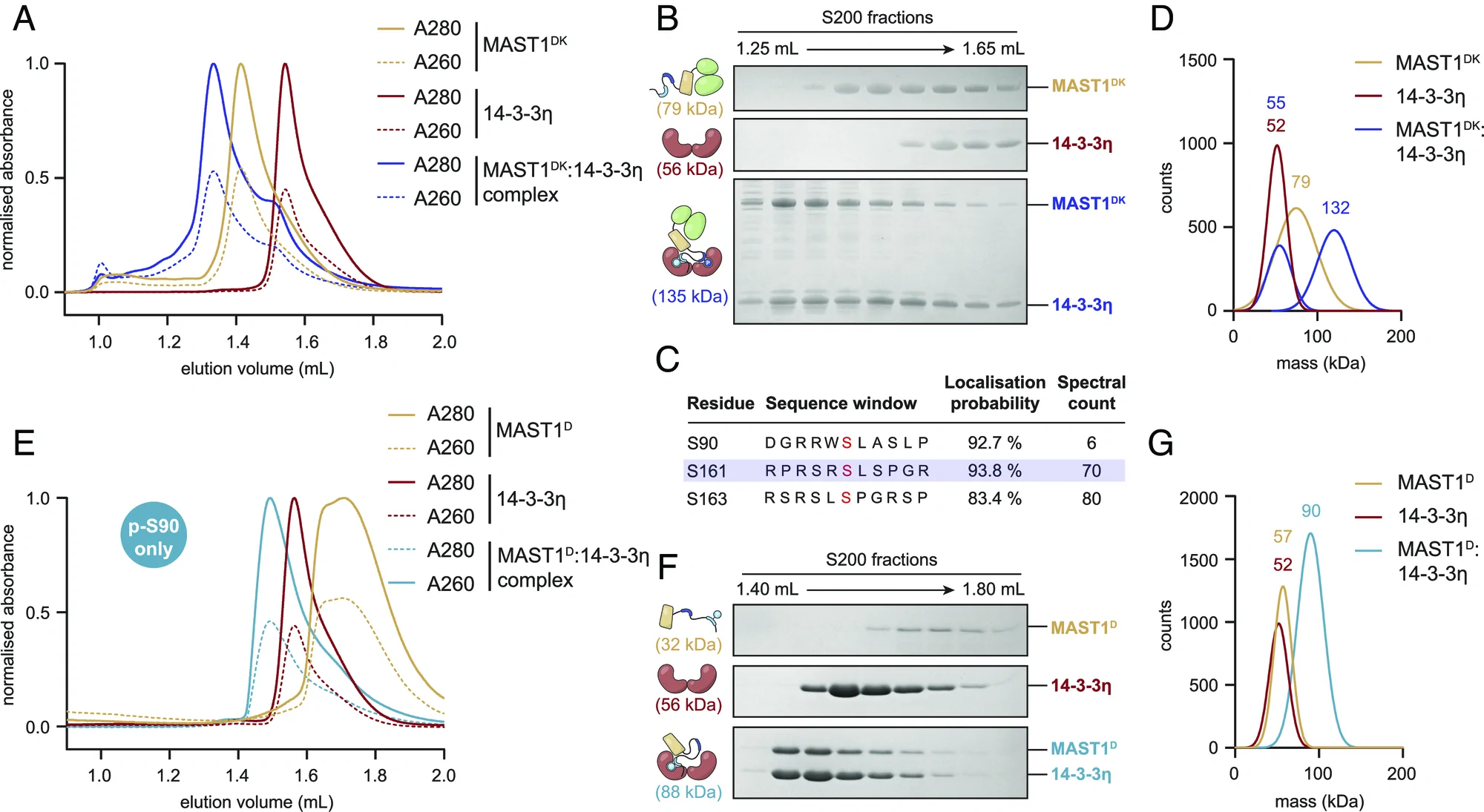
Reconstitution of MAST1:14-3-3η complexes in vitro. (*A*) Analytical SEC elution profiles of purified MAST1^DK^ (yellow), purified 14-3-3η (red), and a copurified complex of MAST1^DK^ and 14-3-3η (dark blue). Absorbance at 280 nm (solid line) and 260 nm (dashed line). (*B*) Coomassie-stained SDS-PAGE gel of SEC fractions spanning elution peaks in panel *A*. Theoretical masses of each protein/complex shown in parentheses. (*C*) Tandem MS analysis of copurified MAST1^DK^ and 14-3-3η complex to determine phosphorylation state of MAST1 in the complex. Phosphorylated serines are indicated in red. S90, S161 (highlighted in dark blue), and S163 sites shown in the table. See Dataset S3 for full data. (*D*) Gaussian fits from mass photometry of purified MAST1^DK^ (yellow), purified 14-3-3η (red), and a copurified complex of MAST1^DK^ and 14-3-3η (dark blue). Experimentally measured masses of each protein/complex indicated above the Gaussian fit. (*E*) Analytical SEC elution profiles of purified MAST1^D^ p-S90 (yellow), purified 14-3-3η (red), and an in vitro-formed complex of MAST1^D^ p-S90 and 14-3-3η (light blue). Absorbance at 280 nm (solid line) and 260 nm (dashed line). (*F*) Coomassie-stained SDS-PAGE gel of SEC fractions spanning elution peaks in panel *E*. Theoretical masses of each protein/complex shown in parentheses. (*G*) Gaussian fits from mass photometry of purified MAST1^D^ p-S90 (yellow), purified 14-3-3η (red), and an in vitro-formed complex of MAST1^D^ p-S90 and 14-3-3η (light blue). Experimentally measured masses of each protein/complex indicated above the Gaussian fit.

Having established that MAST1 interacts specifically with 14-3-3η in a phosphorylation-dependent manner both in cells and in vitro, we sought to reconstitute stable complexes of the purified proteins in vitro. To first reconstitute S90-phosphorylated MAST1:14-3-3η, we coexpressed MAST1^DK^ with 14-3-3η and PAK4^FL^ in baculovirus-infected Sf9 insect cells. Affinity purification of the GST-tagged 14-3-3η yielded a complex with MAST1 which remained stable during size-exclusion chromatography (SEC) (Fig. 3 *A* and *B*). However, tandem mass spectrometry revealed that MAST1 was not only phosphorylated on S90 and S161 but also, unexpectedly, on S163, with higher spectral counts for p-S161 and p-S163 than p-S90 (Fig. 3*C* and Dataset S4) (48). The origin and significance of MAST1 phosphorylation on S163 in the insect cells is unclear, but a phospho-peptide corresponding to the S163 motif is incapable of binding to 14-3-3η (*SI Appendix*, Fig. S8*A*). Therefore, the complex of MAST1^DK^ and 14-3-3η isolated from insect cells coexpressing PAK4 is mediated by phosphorylation of S90, S161, or a combination of both, in line with our observations from AP-MS. To determine the stoichiometry of the complex, we measured the mass of the particles by mass photometry (49). Unfortunately, at the concentrations required for mass photometry, the complex dissociated into its constituent components (*SI Appendix*, Fig. S8*B*). We therefore employed glutaraldehyde cross-linking to stabilize the complex (*SI Appendix*, Fig. S8*C*) and measure its stoichiometry. Particles of MAST1^DK^ and 14-3-3η were observed to have an average mass of 132 kDa, which corresponds to a 1:2 complex (Fig. 3*D* and *SI Appendix*, Fig. S8 *D*–*F*). The 1:2 stoichiometry was further supported by gel densitometry analysis of the complex in Fig. 3*B* (*SI Appendix*, Fig. S8*G*).

We next tested whether S90 phosphorylation alone is sufficient to reconstitute a MAST1:14-3-3η complex in vitro. MAST1^D^ purified in isolation was phosphorylated with PAK4^FL^ and subsequently incubated with purified 14-3-3η at a 1:1 stoichiometry, and assessed by analytical SEC (Fig. 3*E*). Complex formation was evidenced by the increase in hydrodynamic radius of MAST1 and the coelution of the two proteins. Gel densitometry of the coeluting peak again indicated a 1:2 complex (Fig. 3*F* and *SI Appendix*, Fig. S8*H*). This was confirmed by glutaraldehyde cross-linking mass photometry, where particles of S90-phosphorylated MAST1^D^ crosslinked to 14-3-3η were observed to have an average mass of 90 kDa, corresponding to a 1:2 complex (Fig. 3*G* and *SI Appendix*, Fig. S8 *D*, *I*, and *J*). In summary, we could reconstitute copurified and in vitro-assembled MAST1:14-3-3η complexes, mediated by S90 and S161 phosphorylation, and both assemble with a stoichiometry of 1:2.

### MAST1 G519S Mice Exhibit an Enlarged Corpus Callosum.

**Fig. 4.**
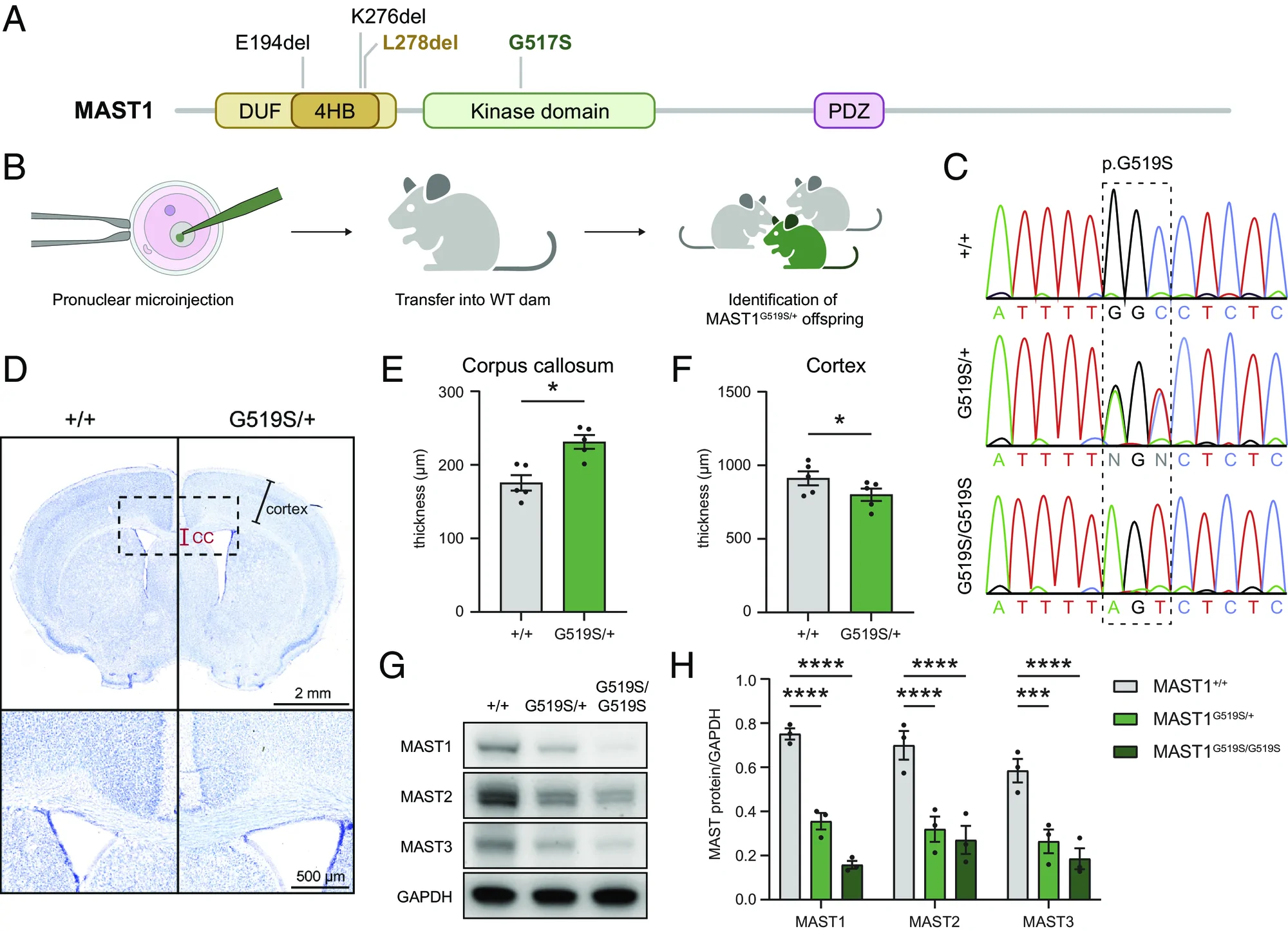
MAST1 G519S mice exhibit an enlarged corpus callosum. (*A*) Schematic of MCC-CH-CM patient-derived mutations in MAST1 protein. G519S (mouse numbering) corresponds to G517S in humans. Mouse models have been generated for the highlighted L278del and G519S variants. (*B*) G519S animals were generated by injection of a single strand DNA repair template and Cas9/guide RNA into the pronucleus of mouse zygotes. Founder animals were identified by Sanger sequencing and backcrossed to C57Bl/6J animals for 10 generations. (*C*) Sequencing traces of MAST1 allele for control (+/+), heterozygous (G519S/+), and homozygous animals (G519S/G519S). (*D*) Nissl stain of coronal 8-wk-old control (+/+) and heterozygous brains (G519S/+) shows an enlargement of the corpus callosum in mutants. Bregma = 0.86 mm. The sites of measurement for corpus callosum (CC) and cortex thickness are indicated in red and black lines respectively. (*E*) Quantification of corpus callosal thickness in control (+/+) and heterozygous brains (G519S/+) (*n* = 5, *P* < 0.05, paired, two-sided *t* test with Bonferroni correction). Data are presented as mean values ± SE. (*F*) Quantification of cortical thickness in control (+/+) and heterozygous brains (G519S/+) (*n* = 5, *P* < 0.05, paired, two-sided *t* test with Bonferroni correction). Data are presented as mean values ± SE. (*G*) Western blot analysis on P0 cortical lysates for MAST1, MAST2, and MAST3 in control (+/+), heterozygous (G519S/+) and homozygous (G519S/G519S) animals. (*H*) Quantification of western blot in panel *F* (*n* = 3, *P < 0.05; **P < 0.01; ***P < 0.001; ****P < 0.0001, two-way ANOVA with Dunnett’s multiple comparisons test). Intensity of bands for MAST family members was normalized to corresponding GAPDH band. Data are presented as mean values ± SE.

To investigate the mechanisms of MCC-CH-CM pathogenicity, we next focused on the molecular consequences of patient-derived MAST1 mutations. MCC-CH-CM patients with heterozygous mutations in MAST1 exhibit gross morphological alterations in their brain structure, with a significantly enlarged corpus callosum (1). Curiously, MAST1 knock-out mice are phenotypically normal (1), implying that the disease is driven by a dominant negative effect of the mutant MAST1 and not by haploinsufficiency. Three of these mutations are triplet deletions in the coding sequence of the 4HB domain and one is a missense mutation in the activation loop of the kinase domain that replaces an invariant glycine in the DFG motif with a serine (Fig. 4*A*). A MAST1^L278del/+^ mouse model has previously been generated and exhibits an enlarged corpus callosum and thinner cortex (1). The G517S patient mutation (G519S in mice) has, as yet, not been phenotypically characterized in a mouse model. Using CRISPR-Cas9 genome editing and subsequent breeding, we generated MAST1^G519S/+^ and MAST1^G519S/G519S^ mice. Following pronuclear injection with guides targeting exon 14 (*SI Appendix*, Table S1), founder animals were identified by Sanger sequencing (Fig. 4 *B* and *C*) and backcrossed for 10 generations to C57Bl/6J. G519S homozygotes died shortly after birth, whereas heterozygotes survived into adulthood. Analysis of adult G519S/+ mouse brains by Nissl staining confirmed the presence of an enlarged corpus callosum with a reduction in cortical thickness (Fig. 4 *D*–*F*) consistent with the disease state and the L278del mouse model. It is worth noting that while the phenotypes of the G519S/+ mouse and the previously described L278del/+ mouse are qualitatively similar, the L278del/+ line displays a more pronounced enlargement of the corpus callosum, hinting toward a more severe molecular disruption caused by the L278del mutation. Analysis of MAST protein levels in P0 cortical samples revealed a dose-dependent decrease in the levels of MAST1, MAST2, and MAST3 in the G519S/+ mouse compared to +/+ (Fig. 4 *G* and *H*) consistent with a dominant negative mechanism. This implies that the disease phenotype stems from a perturbation of MAST signaling in the developing brain.

### Tau Is a Candidate MAST1 Substrate.

**Fig. 5.**
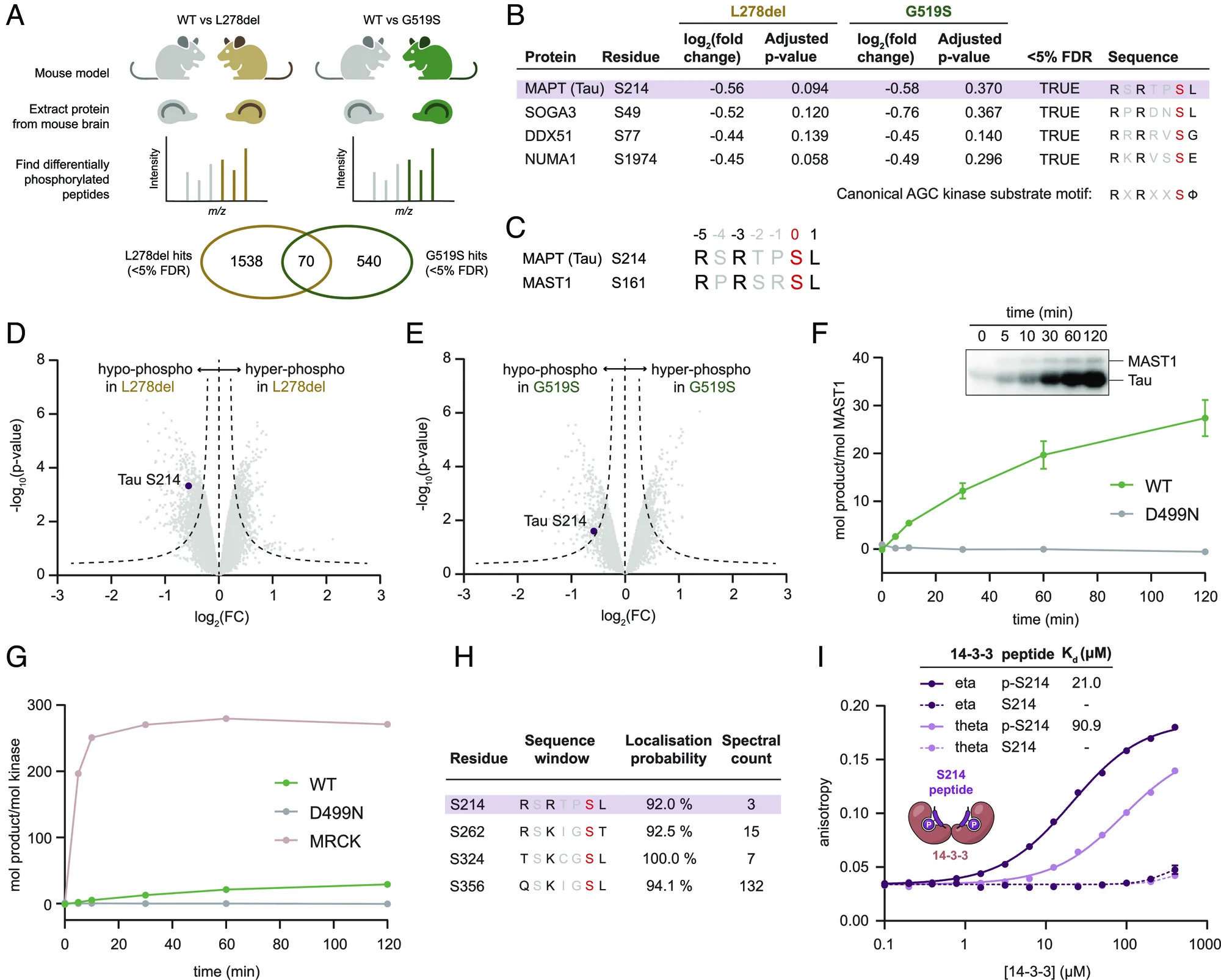
Tau is a candidate MAST1 substrate. (*A*) Schematic of MAST1 WT vs. L278del vs. G519S phosphoproteomics experiment. Number of significantly differentially phosphorylated peptides obtained in each comparison, and shared in both comparisons, shown in Venn diagram [*n* = 5 WT and homozygous litter-matched P0 mice, LIMMA test with 5% FDR threshold described in Hein et al. (43) was applied]. (*B*) Table displaying data from four of the 70 shared phosphosite hits in panel *A*. The four phosphosites listed all conform to the canonical AGC kinase substrate motif (phosphorylated serines indicated in red). MAPT (Tau) is highlighted in purple. (*C*) Sequence alignment of Tau^209-215^ and MAST1^156-162^, where Tau S214 (substrate) and MAST1 S161 (autophosphorylation site) are aligned. (*D*) Volcano plot of phosphoproteomics analysis showing hyperphosphorylated sites (*Right*) and hypophosphorylated sites (*Left*) for L278del when compared to WT. Tau S214 highlighted in purple. Dashed curves represent 5% FDR significance cutoff. (*E*) Volcano plot of phosphoproteomics analysis showing hyperphosphorylated sites (*Right*) and hypophosphorylated sites (*Left*) for G519S when compared to WT. Tau S214 highlighted in purple. Dashed curves represent 5% FDR significance cutoff. (*F*) Radiometric kinase assay by MAST1^DK^ ΔS90 WT or kinase dead (D499N) on Tau. Data are presented as mean values ± SE, *n* = 10 for WT, 3 for D499N. Representative phosphorimage shown in *Inset*. (*G*) Radiometric kinase assay by MRCKα on MLC2, overlaid on assay shown in *F*. (*H*) Tandem MS analysis of Tau after phosphorylation by MAST1^DK^ ΔS90 WT shown in panel *E*. Phosphorylated serines are indicated in red and residues involved in AGC kinase binding (−5, −3, +1) are in black. S214 site (highlighted in purple) as well as three known Tau phosphorylation sites in its repeat domains shown in table. See Dataset S3 for full data. (*I*) Binding curves for 14-3-3η or θ and Tau peptides spanning S214, measured by fluorescence anisotropy. Data are presented as mean values ± SE, *n* = 3. Data were fit with a one-site binding model and the K_d_ derived from curve fitting.

To identify substrates of MAST1, we exploited the previously published MAST1^L278del/L278del^ mouse model and the MAST1^G519S/G519S^ model generated in this study. To explore how the mutations alter the phosphoproteome in the developing brain we extracted protein lysates from P0 cortices, when callosal projection neurons are specified and extending axons to the contralateral hemisphere (50), from both controls and homozygotes for the L278del and G519S lines. A comparative analysis identified 70 phosphopeptides, from 38 proteins, that were observed to be differentially phosphorylated in both L278del and G519S mice when compared to littermate controls (Fig. 5*A* and Dataset S5) (51). By filtering for hypophosphorylated proteins for which the phosphorylated residue was embedded in an AGC kinase consensus motif, we reduced the list of candidate proteins to four (Fig. 5*B*). Of the four candidates, phosphorylation of microtubule-associated protein Tau (MAPT) on S214 was most notable. Tau is a microtubule-associated protein that, like MAST1, is highly expressed in neurons of the developing mouse brain (*SI Appendix*, Fig. S9 *A* and *B*) and the phosphorylation state of S214 is considered to be a biomarker for Alzheimer’s Disease (AD) (52, 53). Inspection of the amino acid sequence surrounding S214 revealed that it is near-identical to the S161 putative autophosphorylation site in MAST1 (Fig. 5*C*). Phosphorylation of Tau on S214 was reduced in both L278del and G519S mice compared to littermate controls (Fig. 5 *D* and *E*).

To assess whether MAST1 can phosphorylate Tau in vitro, we performed a kinase assay with purified, recombinant Tau (mouse isoform F). The purity and monodispersity of Tau^FL^ was confirmed by SEC and mass photometry (*SI Appendix*, Fig. S9 *C* and *D*). Wild-type MAST1^DK^ ΔS90 phosphorylated Tau, and a kinase-dead mutant of MAST1 was completely inactive against Tau (Fig. 5*F*). However, the speed of the reaction was lower than what would be expected of a fully active AGC kinase. To benchmark this quantitatively, we compared MAST1 phosphorylation of Tau to the catalytic activity of the closely related AGC kinase Myotonic Dystrophy Kinase-Related Cdc42-binding Kinase α (MRCKα), on its substrate myosin light chain 2 (MLC2). MRCKα exhibited a 50-fold higher catalytic efficiency than MAST1 (Fig. 5*G*). The posttranslational modification profile of Tau exhibited multiple phosphorylation sites including S214, as well as three known Tau phosphorylation sites (54–56) within its repeat domains (Fig. 5*H* and Dataset S4) (57) but, like MAST1 autophosphorylation (*SI Appendix*, Fig. S7 *H* and *I*), the reaction was very inefficient. To test whether the catalytic activity of MAST1 is increased when in complex with 14-3-3η, we compared Tau phosphorylation by MAST1^DK^ alone or in complex with 14-3-3η but observed no significant increase (*SI Appendix*, Fig. S9*E*).

To investigate whether the low catalytic activity of MAST1 might be related to an inactive conformation of its kinase domain, we inspected the structural model we generated in silico. AlphaFold converges on a high-confidence structure of MAST1 in which the 4HB contacts both the N- and C-lobes of the kinase domain (Fig. 1 *H* and *I* and *SI Appendix*, Fig. S5). The kinase domain is observed to be in the active conformation, as indicated by the formation of a salt bridge between K403 in strand β3 and E422 in helix αC, as well as docking of the hydrophobic motif in the C-tail to the hydrophobic pocket of the N-lobe (*SI Appendix*, Fig. S9*F*). Since the active conformation would be expected to promote robust activity but MAST1 exhibits low activity in vitro, we investigated whether the 4HB domain exerts an autoinhibitory effect on the kinase domain by preventing the conformational dynamics required for substrate phosphorylation (58–60). Curiously, the 4HB domain bridges the N- and C-lobes of the kinase domain via an unusual and exclusively hydrophobic interface comprising residues V421, I425, F428, and A429 of helix αC, L488 and Y491 of helix αE, I493 of the αE-β6 linker, M521 and M524 of the activation loop and V216 and F219 of the 4HB (*SI Appendix*, Fig. S9*F*). The 4HB-kinase domain interface is, nevertheless, entirely compatible with the binding of a substrate peptide in its canonical pose (*SI Appendix*, Fig. S9*F*, mesh). However, the MAST1 kinase domain alone (MAST1^K^) exhibited no detectable catalytic activity against Tau compared to MAST1^DK^ ΔS90 (*SI Appendix*, Fig. S9*G*), suggesting that the role of the 4HB domain is more complex than simple autoinhibition.

Tau phosphorylated on S214 has previously been reported to bind 14-3-3 proteins (61, 62). Given the similarity of the S214 motif in Tau to the S161 motif in MAST1 and the observation that MAST1 phosphorylated on S161 interacts with 14-3-3η, this is not surprising. We confirmed that purified 14-3-3η binds a phosphorylated peptide of the Tau S214 motif by fluorescence anisotropy (Fig. 5*I*). As previously observed for p-S161, the binding was phosphorylation-dependent and 14-3-3η exhibited a higher affinity than 14-3-3θ. Taken together, these findings support a model in which MAST1 activity plays a role in the regulation of the microtubule cytoskeleton of neurons, via Tau.

### MCC-CH-CM Mutations Perturb MAST1 Folding and Kinase Activity.

**Fig. 6.**
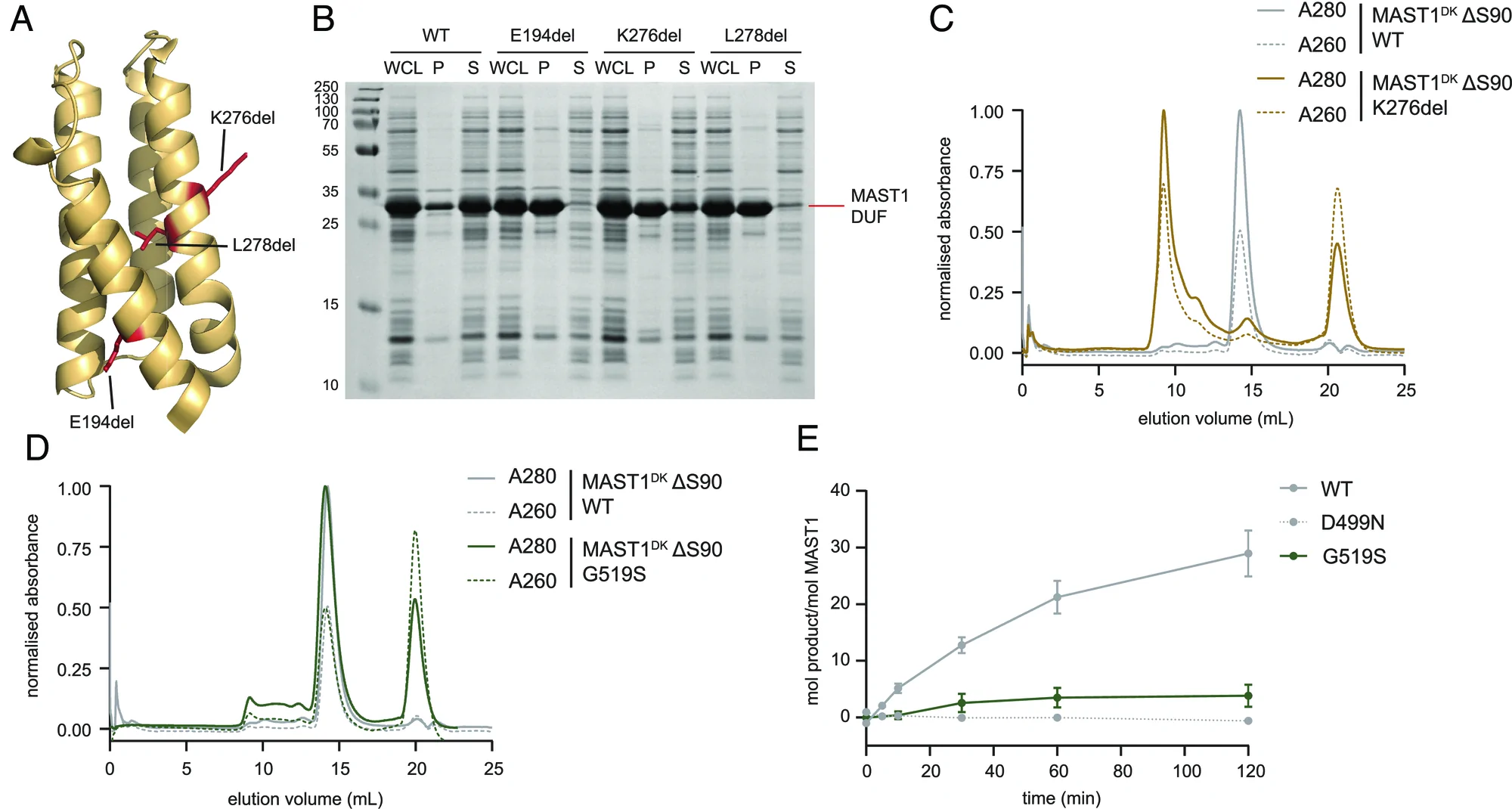
MCC-CH-CM mutations perturb MAST1 folding and kinase activity. (*A*) Structure of MAST1 4HB determined by NMR (PDB ID 2M9X). Patient mutations residing in 4HB highlighted in red and labeled. (*B*) Coomassie-stained SDS-PAGE gel of MAST1^D^ ΔS90 WT, E194del, K276del, or L278del, expressed in *E. coli*. WCL = whole cell lysate, P = pellet, S = supernatant. (*C*) SEC elution profile of purified MAST1^DK^ ΔS90 WT and K276del from Sf9 insect cells, void volume = 9 mL. (*D*) SEC elution profile of purified MAST1^DK^ ΔS90 WT and G519S from Sf9 insect cells. (*E*) Radiometric kinase assay by MAST1^DK^ ΔS90 WT, kinase dead (D499N), or G519S on Tau. Data are presented as mean values ± SE, *n* = 3.

MAST1 proteins in mouse models of patient-derived MCC-CH-CM mutations exert a dominant negative effect by depleting MAST1, 2, and 3 [Fig. 4*H* and Tripathy et al. (1)]. To investigate the molecular basis of pathogenicity, we characterized the effect of the four patient-derived mutations from the original cohort (1) in vitro (Fig. 4*A*). Since E194, K276 and L278 are all located in α-helices of the 4HB domain (Fig. 6*A*), their deletion would be predicted to induce a register shift in the alpha helix that is incompatible with folding of the 4HB domain. We confirmed that deletion of either E194, K276 or L278 leads to misfolding of MAST1^D^ ΔS90 and the formation of insoluble inclusion bodies in *Escherichia coli* (Fig. 6*B*). By contrast, the WT MAST1^D^ ΔS90 is almost entirely soluble (Fig. 6*B* and *SI Appendix*, Fig. S10*A*). Consistently, expression of a longer construct of MAST1 K276del (MAST1^DK^ ΔS90) in Sf9 insect cells resulted in aggregated protein with low yields (Fig. 6*C* and *SI Appendix*, Fig. S10*B*).

Multiple patients have now been identified with a G517S mutation (mouse G519S), in the kinase domain. Although MAST1^DK^ ΔS90 bearing the G519S mutation was properly folded and monomeric by SEC (Fig. 6*D* and *SI Appendix*, Fig. S10*C*), it exhibited significantly lower kinase activity against Tau in vitro, albeit not completely inactive (Fig. 6*E*). Taken together, these observations show that MCC-CH-CM mutations disrupt the folding and/or catalytic activity of MAST1.

## Discussion

**Fig. 7.**
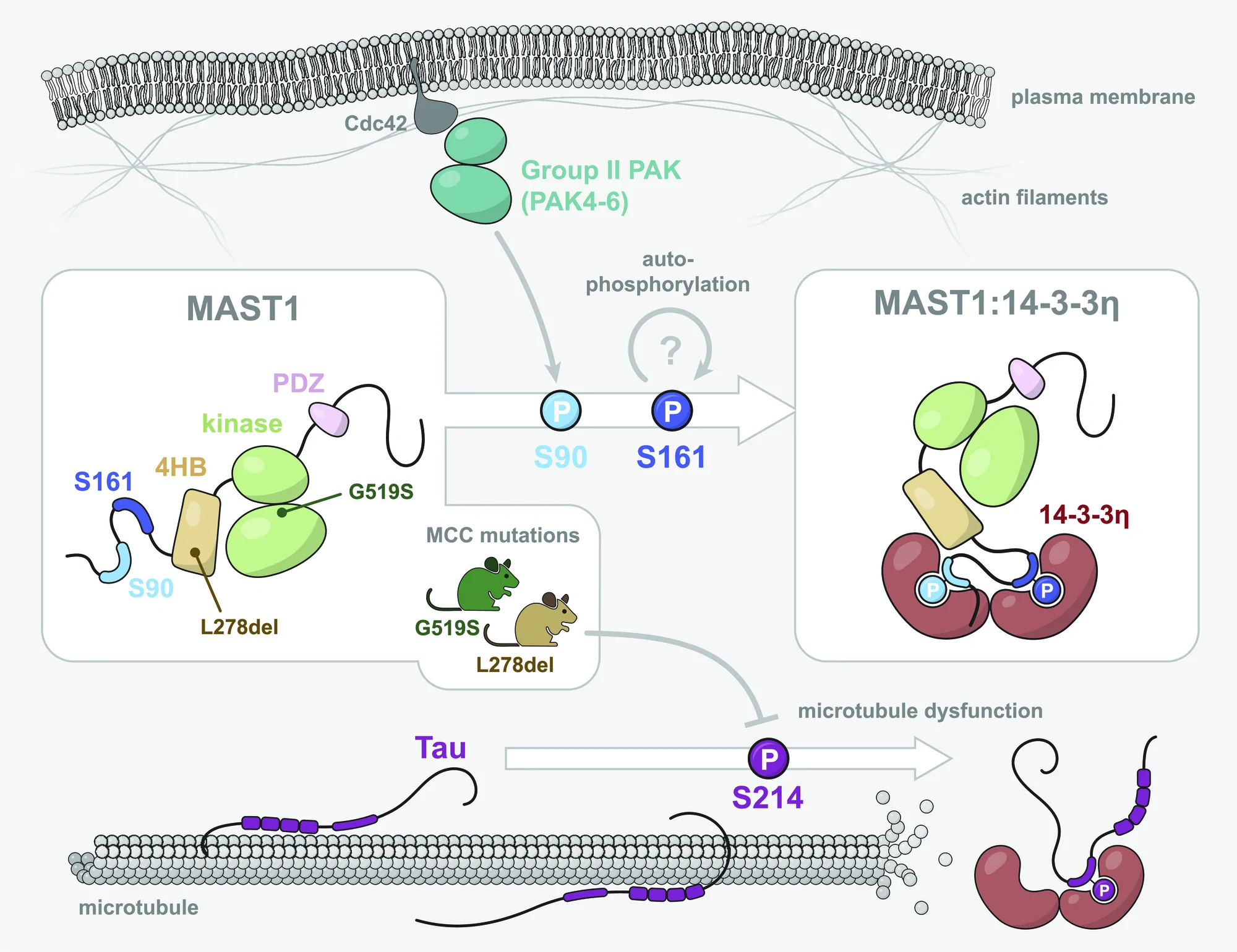
Model of MAST1 regulation and activity. MAST1 has two regulatory phosphosites within its disordered N terminus; S90 (light blue) and S161 (dark blue). A group II PAK (PAK4-6) is activated by Cdc42 at the membrane and phosphorylates MAST1 on S90, while S161 is a putative autophosphorylation site. p-S90 and p-S161 recruit dimeric 14-3-3η and occupy the binding grooves of both protomers, forming a 1:2 trimeric complex of MAST1:14-3-3η. Mouse models harboring MCC-CH-CM mutations in MAST1 (L278del, dark yellow, and G519S, dark green) are hypophosphorylated on Tau S214. Phosphorylation of Tau on S214 results in its dissociation from microtubules and sequestration by 14-3-3, promoting microtubule destabilization. Therefore, MAST1 MCC-CH-CM mutations may cause microtubule dysfunction.

We have found that MAST1 interacts with 14-3-3η in a phosphorylation-specific manner, mediated by S90 and S161. We also identified a group II PAK as the upstream kinase for MAST1 S90 phosphorylation, and S161 as a putative MAST1 autophosphorylation site. Phosphoproteomics to identify candidate MAST1 substrates revealed hypophosphorylation of the microtubule-associated protein Tau in two mouse models of patient-derived MCC-CH-CM mutations. Finally, we provide evidence that Tau is a MAST1 substrate in vitro. Our findings place MAST1 at the interface between the actin and microtubule cytoskeletons in cortical neurons (Fig. 7).

Our finding that PAK phosphorylates MAST1 sheds light on the broader biological pathways that MAST1 may be involved in. PAK4 is highly expressed during embryogenesis, and is crucial in neurodevelopment, where it regulates the proliferation of neural progenitor cells (63), neuronal differentiation, and migration (64). This stems from PAK4’s role in the Cdc42-dependent reorganization of the actin cytoskeleton (65–69). Depletion of either Cdc42 or PAK4 results in cell polarity defects in a number of cell types (70), while knockout of PAK4 is embryonic lethal (64). Both PAK5 and PAK6 activity have been associated with neurite outgrowth (71, 72), while neuronal substrates implicate PAK5 in synaptic vesicle trafficking (73). PAK5/PAK6 double knockout mice are viable, but exhibit locomotor, learning and memory defects (74). Coordination of the actin cytoskeleton is crucial for axonal growth and steering. Actin and microtubule dynamics are coupled in the peripheral domain of the growth cone, together driving accurate axon navigation (75, 76). Importantly, dysregulation of the growth cone can cause axonal rerouting, resulting in the disruption of axonal fiber tracts such as the corpus callosum (77). Here, Cdc42-activated group II PAKs may link actin polymerization and MAST1-mediated microtubule reorganization (Fig. 7). The mechanisms that mediate the colocalization of MAST1 and PAK will require further investigation, but the PDZ domain of MAST1 is likely to play a significant role.

We have identified S214 of Tau as a candidate MAST1 substrate. Tau binds to microtubules via its microtubule binding domain, encompassing the proline-rich domain (which contains S214) and the repeat domains, allowing Tau to rigidify and stabilize microtubules. Several studies have demonstrated the decreased microtubule binding capacity of Tau p-S214, reducing the stability of axonal microtubules (53, 78, 79). This effect is a combination of a decrease in microtubule binding affinity when Tau is phosphorylated (78), and the sequestration of Tau p-S214 by 14-3-3, which we report here and others have previously observed (61, 62). It is also worth noting that, since MAST1 S161 and Tau S214 motifs are near-identical, it is perhaps not surprising that 14-3-3η is a common component in the signaling pathway. Interestingly, S214 has previously been identified as a cAMP-dependent protein kinase (PKA) phosphorylation site (52, 53, 61, 62, 80). Both PKA and the MAST kinases are AGC kinases, which recognize a conserved substrate consensus motif. It is therefore possible that MAST1 and PKA converge on the same substrate under different cellular conditions. Alternatively, the observed Tau S214 hypophosphorylation may reflect an indirect effect on neuronal wiring in the MAST1 mutant mice. In this sense, further work will be required to investigate if Tau is a direct substrate of MAST1 in vivo.

Curiously, hyperphosphorylation of S214 is also a marker of Alzheimer’s disease (52, 53), though the significance of the connection to neurodevelopment is unclear. Our data demonstrate that at least in vitro, MAST1 is capable of phosphorylating Tau on S262, S324 and S356, all of which are reported phosphosites that inhibit microtubule binding (54–56). Nevertheless, these sites deviate from canonical AGC recognition motifs, so whether MAST1 is the physiologically relevant kinase for these sites in vivo will require further investigation. While we have not yet explored the implications of MAST1-mediated Tau phosphorylation in the context of neurodevelopment, we hypothesize that MAST1 may negatively regulate microtubule polymerization in neuronal axons to ensure proper formation of the corpus callosum. Indeed, while the loss of Tau alone appears to have little impact on corpus callosum formation, a Tau and MAP1B double-knockout in mice causes a severe reduction in callosal size (81), beyond the more moderate agenesis phenotype observed in MAP1B-only knockout animals (81, 82). This suggests a potential role of Tau in corpus callosum development, which may be partially redundant with MAP1B.

While our findings imply a PAK-dependent coupling between actin and microtubule reorganization via MAST1, there are still many unanswered questions, particularly regarding the mechanism of activation of MAST1. Our data currently do not support a direct role for 14-3-3η in kinase activation, and MAST1 exhibits low activity in vitro. These observations beg the obvious question of precisely what role 14-3-3η plays in MAST1 regulation. Like MAST1, the oncoprotein BRAF possesses two regulatory phosphoserines, which simultaneously occupy the binding grooves of a 14-3-3 dimer. This trimeric complex maintains BRAF in an inactive conformation (37, 83), which, upon RAS binding, is converted into an active complex with 2:2 stoichiometry (37). While it is easy to envisage a similar mode of regulation for MAST1, both p-S90 and p-S161-mediated MAST1:14-3-3η complexes were observed to be trimeric and a tetrameric complex appears to be sterically improbable.

The low catalytic activity of recombinant MAST1 suggests that there may be another, as yet unidentified, factor that drives its activation. This is particularly relevant for the identification of the S161 kinase, which we suggest might be MAST1 itself. In vitro, MAST1 exhibits low and nonspecific activity against both S161 and Tau S214, suggesting that MAST1 is not fully active. While in silico modeling suggests that the 4HB domain might exert an inhibitory influence on the kinase domain, its deletion did not activate MAST1. This implies that the kinase domain alone is not sufficient for MAST1 activity and that other factors must regulate it. However, we cannot exclude the possibility that neither site is a bona fide MAST1 substrate. Determining what is necessary and sufficient for full MAST1 activation will be important to decipher the biological processes that the MAST kinases regulate.

Finally, this study provides important insights into the molecular consequences of MCC-CH-CM disease-associated mutations. We show that pathogenic mutations in MAST1 perturb folding and/or catalytic activity of the protein. Similar to the L278del mouse mutant (1), we report that the G519S mutation causes a notable decrease in the protein levels of MAST2, MAST3 and MAST4. Since MAST1 knock-out mice are phenotypically normal, this implies that MCC-CH-CM is the consequence of a dominant negative effect. However, the observed 1:2 MAST1:14-3-3 stoichiometry precludes that the dominant negative effect is mediated by 14-3-3 dimers. Instead, the integration of MAST kinases into homo- or heteromeric complexes mediated by other factors is more likely to underpin the phenotype. Alternatively, the localization of mutant MAST1 to microtubules or titration of essential cofactors may inhibit signaling by wild-type MAST1.

In summary, MAST1 is a key regulator of neurodevelopment that potentially links group II PAKs, neuronal regulators of the actin cytoskeleton, to microtubule dynamics. However, several questions surrounding the precise mechanism of MAST1 activation, the origin of the dominant negative effect, and the role of 14-3-3η in MAST1 regulation remain. Answers to these questions are undoubtedly required to elucidate the physiological role of MAST1 in neurodevelopment as well as its pathological role in MCC-CH-CM.

## Materials and Methods

### EGFP-MAST1 AP-MS.

N-terminally EGFP-tagged MAST1^DKP^ constructs (WT, ΔS90, ΔS90 ΔS161, S90A, S161A, S90A S161A) were cloned into the pEGFP-C1 vector for expression in HEK293 cells and were transfected using Lipofectamine 3000 (Invitrogen). Media on the cells was exchanged 24 h posttransfection and cells were harvested by trypsinization 48 h posttransfection. Cells were lysed with three freeze/thaw cycles in liquid nitrogen, resuspended in lysis buffer (10 mM monosodium phosphate, 40 mM disodium phosphate, 10 mM NaCl, 50 mM NaF, 1 mM TCEP, 10 U Denarase, 1× protease inhibitor cocktail (Sigma P8849), 2 mM benzamidine, 2 mM MgCl2, 0.25% CHAPS, 10 mM betaglycerophosphate, 2 mM sodium pyrophosphate, 1 mM sodium orthovanadate, 1 mM EDTA), and incubated at 4 °C for 30 min with rotation. Cleared lysates were obtained by centrifuging at 21,000 g, 4 °C, for 15 min. Cleared lysates were incubated with GFP-Trap Magnetic beads (Chromotek), incubated at 4 °C for 2 h while rotating, washed five times with wash buffer (10 mM monosodium phosphate, 40 mM disodium phosphate, 10 mM NaCl, 50 mM NaF, 1 mM TCEP), and the dry beads were snap-frozen in liquid nitrogen and stored at −80 °C, for analysis by mass spectrometry.

### In Silico AlphaFold Modeling.

AlphaFold 3 was implemented in two steps: curation of MAST sequences and alignment using MAFFT, followed by structure inference using the MSA as input. For sequence curation we searched UniProt for sequences containing the InterPro domain identifiers IPR050236 or IPR000961 (KD), IPR015022 or IPR023142 (4HB), and IPR001478, IPR041489, or IPR036034 (PDZ), respectively. We aligned the sequences using MAFFT and taxonomically balanced and downsampled the alignment to 1,000 sequences according to Krötenheerdt et al. (36). The same procedure was followed to generate an alignment of 14-3-3 sequences. The resulting MSAs were fed into AlphaFold 3 (27) to generate an input json file for structure inference, which was then used to predict 100 models with independent starting poses (100 model seeds). The resulting models were superimposed on each other using the backbone coordinates of the 14-3-3 chains and the arrangement of the respective proteins and domains compared by Euclidean geometry as described in ref. 36.

### Fluorescence Anisotropy.

A 2× serial dilution series was made with purified protein (14-3-3η, 14-3-3θ, or MAST1^DK^ ΔS90 ΔS161) and mixed with 50 nM of N-terminally FITC-conjugated peptide and incubated at room temperature for 15 min. The following peptides were used in this study: DGRRWSLASLPSS (MAST1^85-97^), DGRRW{pS}LASLPSS (MAST1^85-97^ p-S90), RPRSRSLSPGRSP (MAST1^156-168^), RPRSR{pS}LSPGRSP (MAST1^156-168^ p-S161), PLGDP{pS}GRWARLS (MAST1 p-Scramble), RSRSL{pS}PGRSPSS (MAST1^158-170^ p-S163), PAVRPRSRSLSPGR (MAST1^153-166^ WT), PAVEPESRSLSPGR (MAST1^153-166^ R156E R158E), PAVEPESRSDSPGR (MAST1^153-166^ R156E R158E L162D), RSRTPSLPTPPTR (Tau^209-221^), and RSRTP{pS}LPTPPTR (Tau^209-221^ p-S214). Anisotropy was measured in a 384-well black polystyrene plate on the TECAN Spark Multi-Mode Microplate Reader.

### Radiometric Kinase Assay.

For substrate phosphorylation assays, 100 nM of kinase was mixed with 25 μM of substrate in assay buffer (50 mM HEPES pH 7.4, 150 mM NaCl, 1 mM TCEP, 2 mM MgCl_2_, 2 mM ATP, 0.25% CHAPS, spiked with 1 μL [γ-32P] ATP/100 μL reaction) and incubated at 23 °C for up to 2 h. Aliquots were taken from the reaction volume and quenched with 80 mM EDTA over a time course, then equal volumes of each sample were subjected to SDS-PAGE. The gels were washed three times in dH_2_0, dried, exposed to a phosphor screen for 4 h, and imaged with an Amersham Typhoon imager (Cytiva). Densitometry was performed using ImageJ to quantify the bands of phosphorylated substrate. An internal standard was made by spotting 0.5, 1, 5, and 20 pmol of ATP from the reaction onto a strip of Whatman paper. The standards were also measured with densitometry and used to calculate the total mol of phosphorylated substrate in each sample.

For autophosphorylation assays, 50 nM, 150 nM, and 500 nM of MAST1^DK^ ΔS90 (WT and D499N) was incubated in assay buffer (50 mM HEPES pH 7.4, 150 mM NaCl, 1 mM TCEP, 2 mM MgCl_2_, 2 mM ATP, 0.25% CHAPS, spiked with 1 μL [γ-32P] ATP/100 μL reaction) at 23 °C for up to 2 h. Aliquots were taken from the reaction volume and quenched with 80 mM EDTA over a time course, then equal volumes of each sample were spotted onto a 0.45 μm nitrocellulose membrane. The membrane was washed five times with 20 mL of 75 mM H_3_PO_4_, and exposed and imaged as described above. An internal standard and densitometry were performed as described above.

### Analytical Size Exclusion Chromatography.

A Superdex 200 Increase 3.2/300 size exclusion column (Cytiva) was connected to an Äkta Pure with a Micro configuration (Cytiva) and equilibrated in buffer (50 mM HEPES pH 7.4, 150 mM NaCl, 1 mM TCEP) at 4 °C. 50 μL of 30 μM protein samples were centrifuged at 21,000 g for 5 min prior to injection onto the column. For the MAST1^D^ p-S90 and 14-3-3η complex formed in vitro, equimolar concentrations of each protein were mixed and incubated for 30 min at room temperature prior to injection. 50 μL fractions were collected from each run for analysis by Coomassie-stained SDS-PAGE.

### Mass Photometry.

Purified protein samples were clarified after thawing by centrifugation at 21,000 g, 4 °C for 5 min and diluted to 0.5 μM in freshly filtered buffer (50 mM HEPES pH 7.4, 150 mM NaCl, 1 mM TCEP). Measurements of 60 s duration were recorded at a final concentration of 25 to 50 nM on microscopy coverslips that were cleaned by sonication in water and isopropanol. All measurements were recorded using a Two^MP^ mass photometer (Refeyn) and analyzed using Discover MP (Refeyn). Protein mass was calculated using a contrast-to-mass calibration with NativeMark Protein Standard (Invitrogen).

### Histological Analysis of Mice.

Adult brains were isolated after transcardial perfusion with 0.9% NaCl followed by 4% PFA for 5 min at a flow rate of approximately 6 mL per minute. Brains were dissected and postfixed in 4% PFA overnight at 4 °C. Brains were washed with PBS and cryoprotected in 30% Sucrose in PBS. For sectioning, brains were embedded in Neg-50™ Medium (Richard-Allan Scientific) and sectioned into 40 µM-thick coronal brain sections using a sledge microtome. Nissl stainings were carried out via incubation in a 0.25% solution of Cresyl Violet acetate (Sigma, #C5042), followed by dehydration in a graded ethanol series (30%, 70%, 95%, 100%; 3 min, 2 min, 30 s, 10 s) and xylol (2 × 2 min) and mounted using DPX mountant (Fluka, #44581). Slides were left to solidify overnight at RT and images were acquired using a Mirax slide scanner (Zeiss). The thickness of the corpus callosum was measured at Bregma + 0.86 mm by performing 3 parallel measurements near the midline position using ImageJ. The cortical thickness was measured at Bregma −1.82 mm through bilateral measurements of the somatosensory cortex at 3 matched positions. Averages were taken and used for analysis.

## Supplementary Material

Appendix 01 (PDF)

Dataset S01 (XLSX)

Dataset S02 (XLSX)

Dataset S03 (XLSX)

Dataset S04 (XLSX)

Dataset S05 (XLSX)

## Data Availability

Mass spectrometry data have been deposited in PRIDE (PXD063727, PXD063803, PXD063802, PXD063795, PXD063731, PXD080824, and PXD065759) (20, 28, 45, 46, 48, 51, 57).
