# Supplementary material for: A complex of MAST1 and 14-3-3η regulates Tau phosphorylation in the developing cortex
Source: Proc Natl Acad Sci U S A. 2026 Sep 17;123(38):e2617534123. doi: 10.1073/pnas.2617534123 (PMC13598245; doi:10.1073/pnas.2617534123)
Supplement: Supplementary file 1 [file pnas.2617534123.sapp.pdf]

Supplementary Information

A complex of MAST1 and 14-3-3η regulates Tau phosphorylation in the developing cortex

Sumire Antonioli[1,2,§], Patrick Heisterkamp[3,§], Weiqiang Chen[4], Dorothea Anrather[4], Markus Hartl[4,5], Maria-Fernanda Martinez-Reza[6], Ratna Tripathy[7], Michael Schutzbier[7], Karl Mechtler[7], David A. Keays[3,7] and Thomas A. Leonard[1*]

[1]Max Perutz Labs, Medical University of Vienna, Dr. Bohr-Gasse 9, 1030, Vienna, Austria.

[2]Vienna BioCenter PhD Program, a Doctoral School of the University of Vienna and the Medical University of Vienna, A-1030 Vienna, Austria.

[3]Division of Neurobiology, Department of Biology, Ludwig-Maximilians-University Munich, Grosshaderner Str. 2, 82152 Planegg-Martinsried, Germany

[4]Max Perutz Labs, Mass Spectrometry Facility, Vienna Biocenter Campus (VBC), Dr. Bohr-Gasse 9, 1030, Vienna, Austria.

[5]University of Vienna, Center for Molecular Biology, Department of Biochemistry and Cell Biology, Dr. Bohr-Gasse 9, 1030, Vienna, Austria.

[6]Faculty of Medicine, Ludwig-Maximilians-University Munich, Grosshaderner Str. 9, 82152 Planegg-Martinsried, Germany

[7] Institute of Molecular Pathology (IMP), Dr-Bohr Gasse 7, Vienna 1030, Vienna Biocentre (VBC), Austria.

[§]These authors contributed equally to this work.

[*]Correspondence: thomas.leonard@meduniwien.ac.at

Contains: Supplementary Figures 1-10

Supplementary Table 1

Supplementary Files 1-5 Legends

Supplementary Methods

Supplementary References

Supplementary Figures

Figure S1. MAST1 interacts with 14-3-3 proteins via S90 and S161.

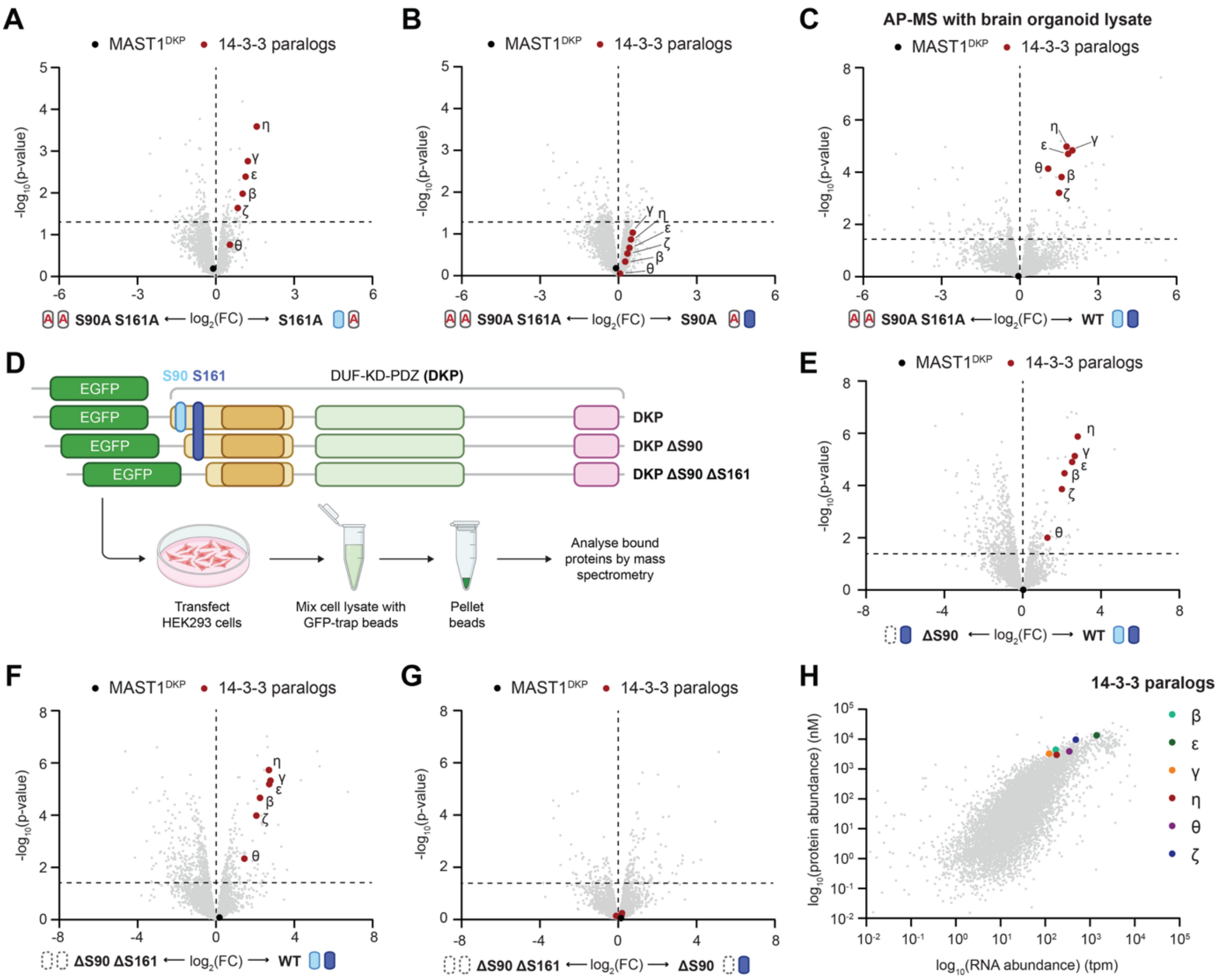

A. Volcano plot displaying the enriched interactome of EGFP-MAST1^{DKP} S161A (right) vs EGFP-MAST1^{DKP} S90A S161A (left) from AP-MS, *n* = 3. Horizontal dashed line represents significance cut-off ($p < 0.05$).

B. Volcano plot displaying the enriched interactome of EGFP-MAST1^{DKP} S90A (right) vs EGFP-MAST1^{DKP} S90A S161A (left) from AP-MS, *n* = 3. Horizontal dashed line represents significance cut-off ($p < 0.05$).

C. Volcano plot displaying the enriched interactome of EGFP-MAST1^{DKP} (right) vs EGFP-MAST1^{DKP} S90A S161A (left) from AP-MS with day 120 dorsal brain organoid lysates, *n = 3*. Horizontal dashed line represents significance cut-off ($p < 0.05$).

D. Construct boundaries and experimental setup for EGFP-MAST1^{DKP} AP-MS in HEK293 cells.

E. Volcano plot displaying the enriched interactome of EGFP-MAST1^{DKP} (right) vs EGFP-MAST1^{DKP} ΔS90 (left) from AP-MS, *n* = 3. Horizontal dashed line represents significance cut-off ($p < 0.05$).

F. Volcano plot displaying the enriched interactome of EGFP-MAST1^{DKP} (right) vs EGFP-MAST1^{DKP} ΔS90 ΔS161 (left) from AP-MS, *n* = 3. Horizontal dashed line represents significance cut-off ($p < 0.05$).

G. Volcano plot displaying the enriched interactome of EGFP-MAST1^{DKP} ΔS90 (right) vs EGFP-MAST1^{DKP} ΔS90 ΔS161 (left) from AP-MS, *n* = 3. Horizontal dashed line represents significance cut-off ($p < 0.05$).

H. Scatter plot showing RNA and protein abundance of all six 14-3-3 paralogs identified in Figure 1C (highlighted by large colored dots) overlayed on all detected RNA/protein, in HEK293 cells (1).

Figure S2. MAST1 and 14-3-3η are co-expressed in developing cortical neurons.

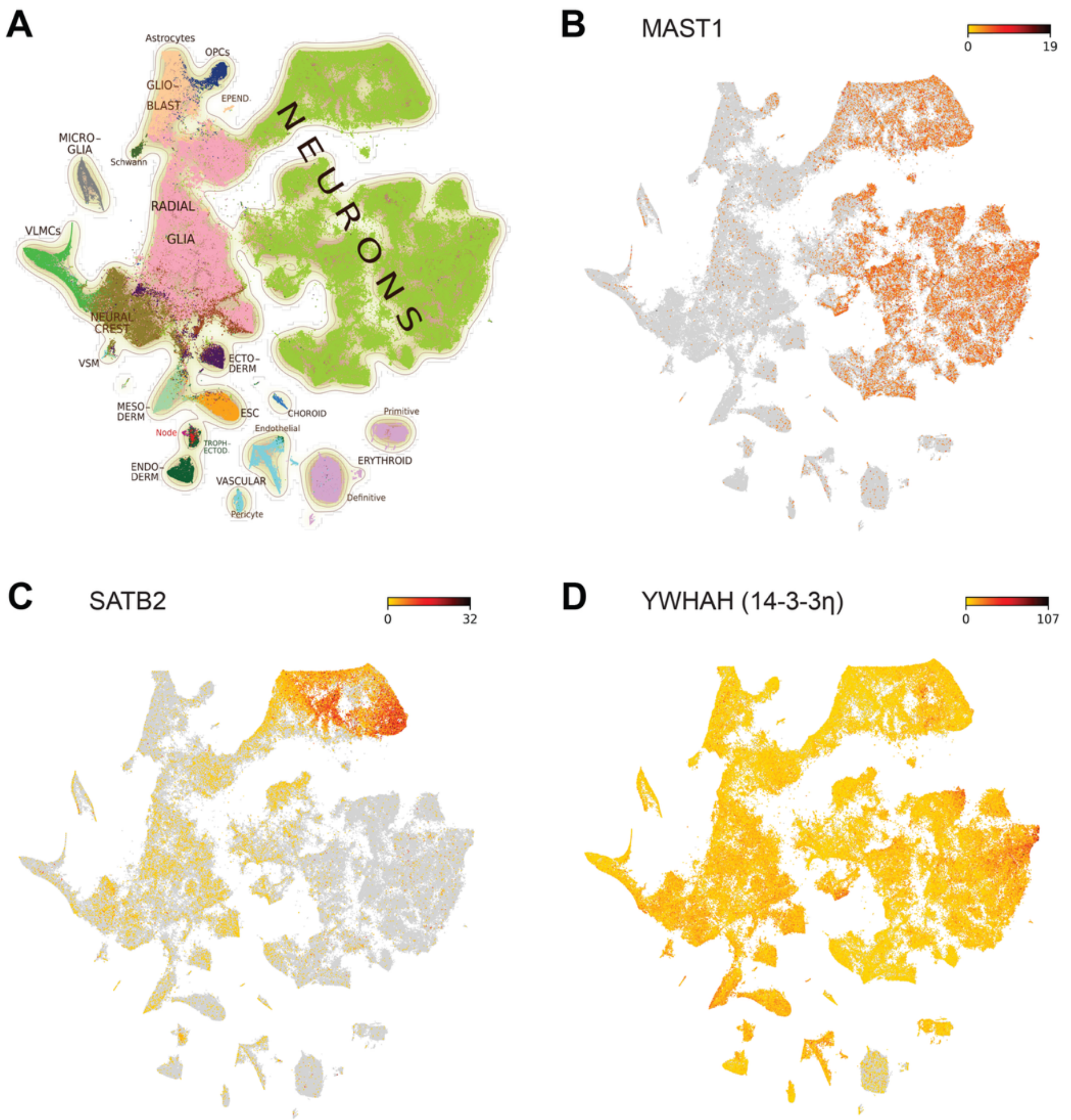

A. t-SNE plot of single-cell RNA sequencing data from embryonic mouse brains between embryonic stage E7-E18, colored by major cell types (2).

B. MAST1 expression levels overlayed on t-SNE plot from panel A.

C. SATB2 expression levels overlayed on t-SNE plot from panel A.

D. YWHAH (14-3-3η) expression levels overlayed on t-SNE plot from panel A.

Figure S3. Mass spectra of all protein constructs employed in this study.

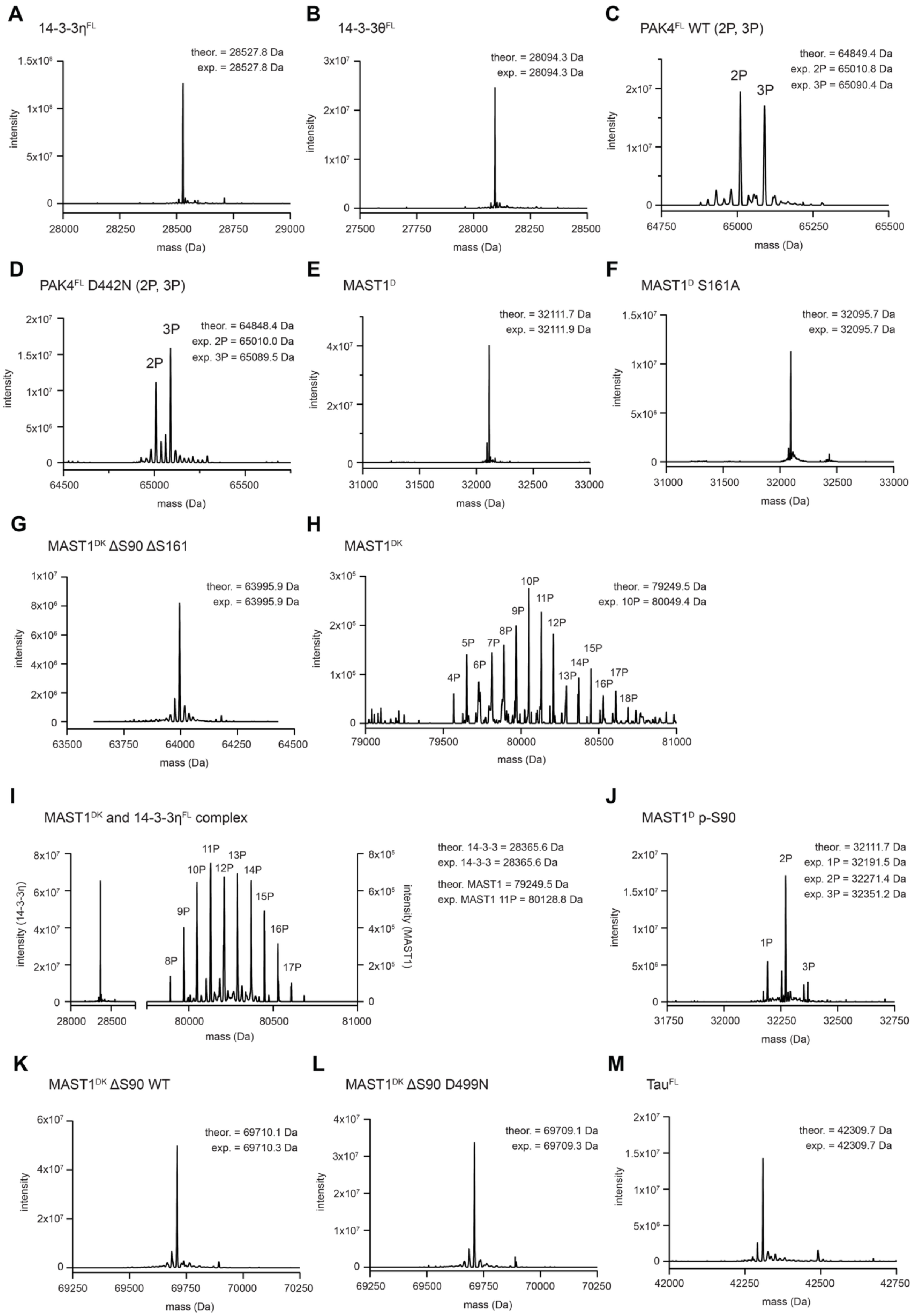

A. Mass spectrum of purified 14-3-3η^{FL}.

B. Mass spectrum of purified 14-3-3θ^{FL}.

C. Mass spectrum of purified PAK4^{FL} WT.

D. Mass spectrum of purified PAK4^{FL} D442N.

E. Mass spectrum of purified MAST1^{D}.

F. Mass spectrum of purified MAST1^{D} S161A.

G. Mass spectrum of purified and dephosphorylated MAST1^{DK} ΔS90 ΔS161.

H. Mass spectrum of purified MAST1^{DK}.

I. Mass spectrum of co-purified MAST1^{DK} and 14-3-3η^{FL} complex.

J. Mass spectrum of purified MAST1^{D} after phosphorylation by PAK4^{FL} WT.

K. Mass spectrum of purified and dephosphorylated MAST1^{DK} ΔS90 WT.

L. Mass spectrum of purified and dephosphorylated MAST1^{DK} ΔS90 D499N.

M. Mass spectrum of purified Tau^{FL}.

Figure S4. Mass spectra of all protein constructs employed in this study (continued).

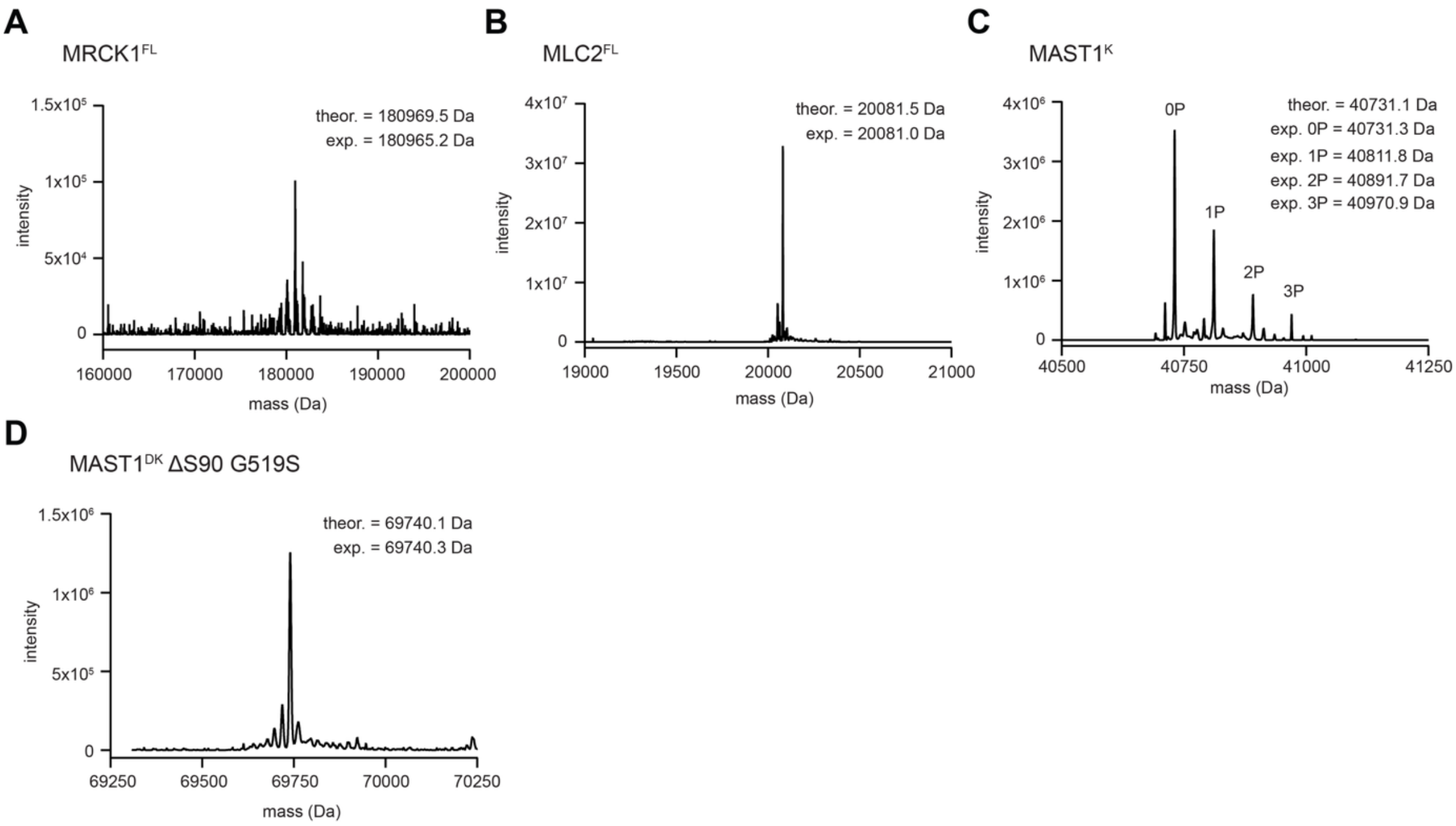

A. Mass spectrum of purified MRCK1^{FL}.

B. Mass spectrum of purified MLC2^{FL}.

C. Mass spectrum of purified MAST1^{K}.

D. Mass spectrum of purified and dephosphorylated MAST1^{DK} ΔS90 G519S.

Figure S5. AlphaFold modeling of MAST1:14-3-3η complex.

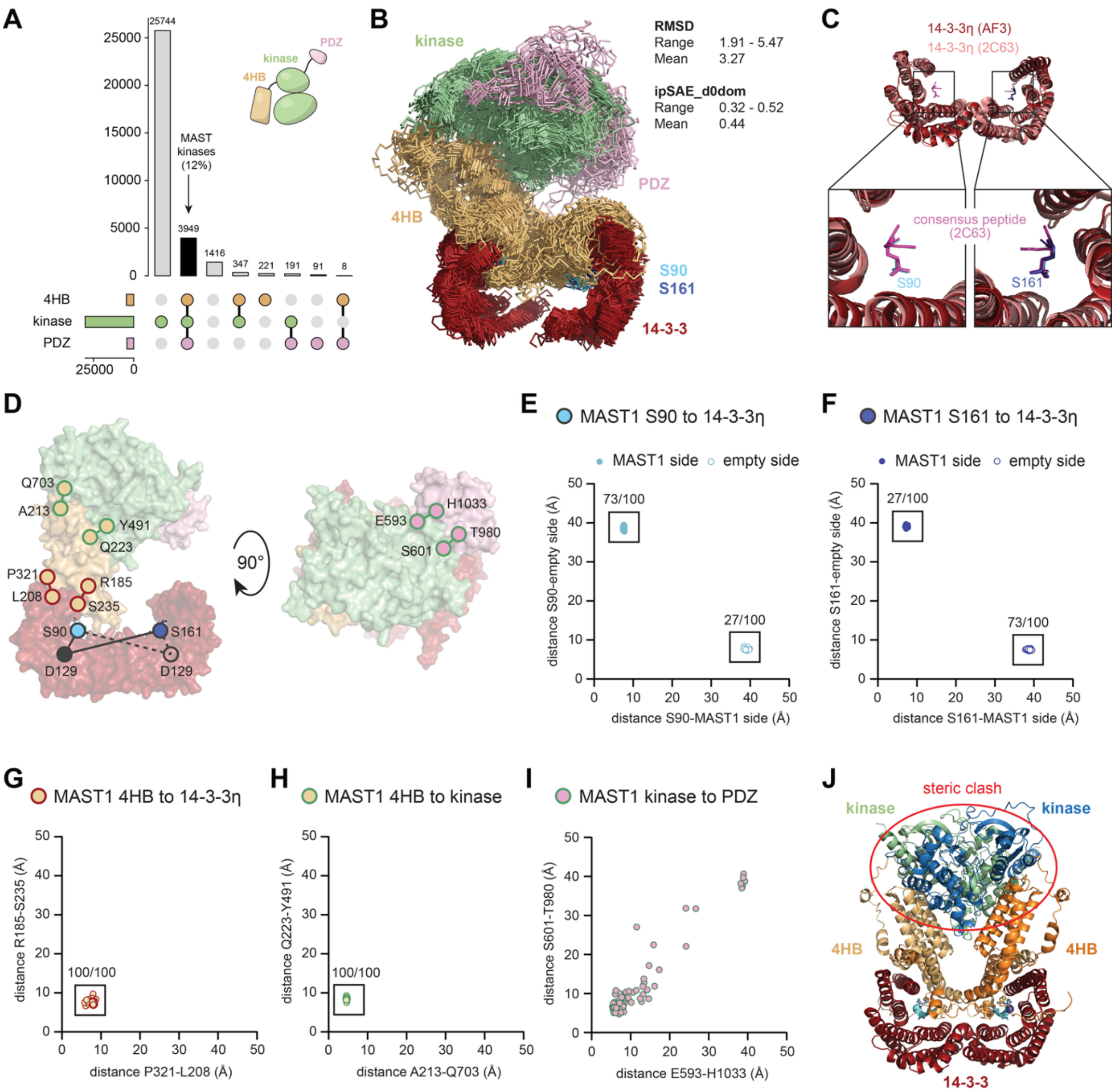

A. UpSet plot of 31967 sequences in the MSA curated by AlphaFold3 for full length MAST1 prediction. Partial sequences were mapped back to their parental full-length sequences, which were then binned according to the presence or absence of InterPro domains corresponding to the 4HB (IPR015022 or IPR023142, yellow), kinase domain (IPR050236 or IPR000961, green), and PDZ domain (IPR001478, IPR041489 or IPR036034, pink) of MAST kinases. 3949 sequences contained all three MAST domains, representing 12% of the MSA (highlighted in black).

B. Superposition of 100 independent AlphaFold3 models of MAST1 and 14-3-3η in a 1:2 stoichiometry, using a curated, taxonomically balanced MSA containing only MAST sequences (3). Only MAST1 residues 70-724 and 965-1064 shown for clarity. RMSD between models was calculated over Cα atoms of MAST1 168-724, ipSAE_d0dom is reported for the interface between the 4HB and the 14-3-3η protomer closest to MAST1 for each model (4).

C. Superposition of 14-3-3η from one representative MAST1:14-3-3η AlphaFold model (red) with an experimentally determined crystal structure of 14-3-3η in complex with a ligand phosphopeptide (salmon, PDB ID 2C63) (5). p-S90, p-S161, and ligand phosphopeptide highlighted in light blue, dark blue, and pink, respectively. RMSD = 1.072 (calculated over Cα atoms of both 14-3-3 chains).

D. Residue pairs chosen between domain interfaces for Euclidean distance analysis. Pairs are linked with a line and colored according to the domains that they bridge. 14-3-3 binding grooves closest to and furthest from MAST1 are indicated with a grey closed and open circle, respectively.

E. Euclidean distance between the phosphorus of S90 gamma phosphate and Cα of D129 from both 14-3-3 protomers, for 100 AlphaFold 3 models. S90 binds on the side closest to MAST1 in 73/100 models.

F. Euclidean distance between the phosphorus of S161 gamma phosphate and Cα of D129 from both 14-3-3 protomers, for 100 AlphaFold 3 models. S161 binds on the side closest to MAST1 in 27/100 models.

G. Euclidean distance between the Cα of both 4HB and 14-3-3η residue pairs, for 100 AlphaFold 3 models.

H. Euclidean distance between the Cα of both 4HB and kinase domain residue pairs, for 100 AlphaFold 3 models.

I. Euclidean distance between the Cα of both kinase domain and PDZ domain residue pairs, for 100 AlphaFold 3 models.

J. Superposition of one representative MAST1:14-3-3η AlphaFold model flipped along its vertical axis, representing a complex with 2:2 stoichiometry. Steric clashes between the two kinase domains are circled in red.

Figure S6. PAK4 phosphorylates MAST1 on S90.

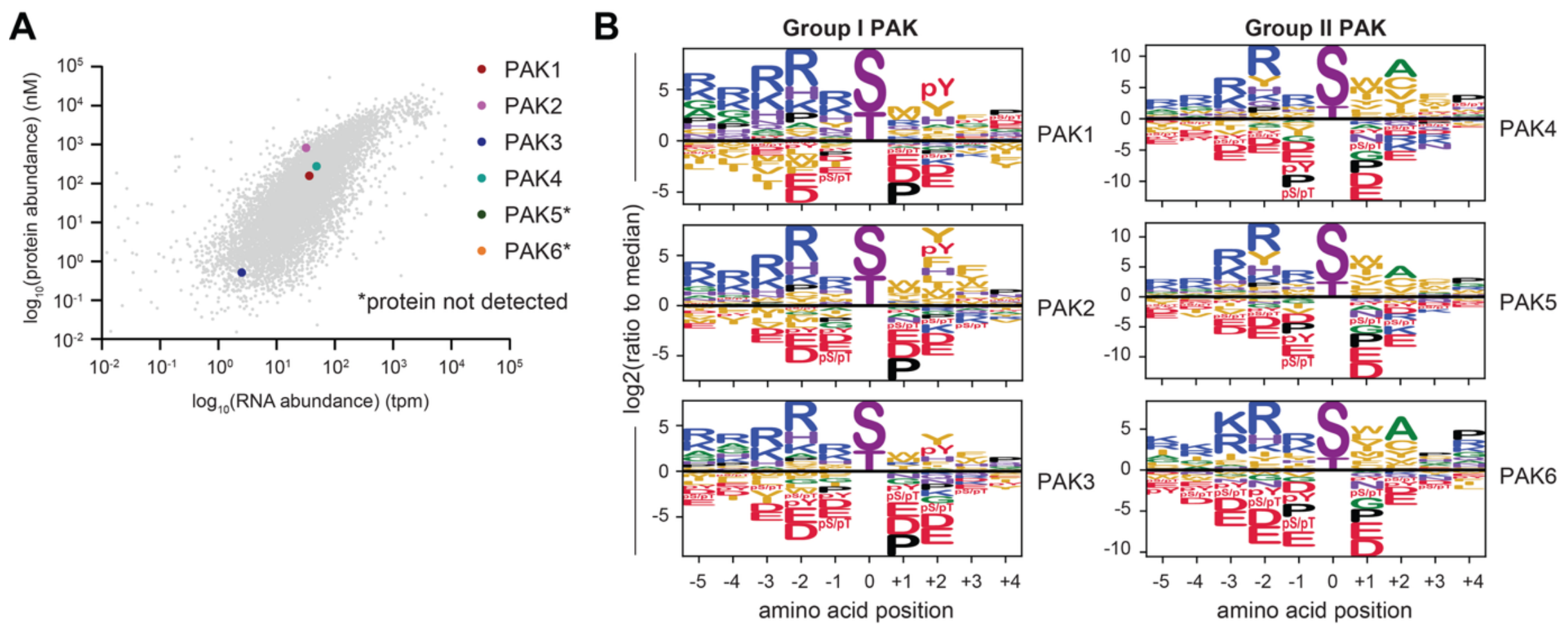

A. Scatter plot showing RNA and protein abundance of all PAKs (highlighted by large colored dots) overlayed on all detected RNA/protein, in HEK293 cells (1). Both PAK5 and PAK6 protein was not detected, and are not plotted.

B. Substrate motif sequence logos of group I PAKs (PAK1-3) and group II PAKs (PAK4-6) from P-5 to P+4, where position 0 is the phosphorylatable residue (6).

Figure S7. MAST1 S161 is a putative autoregulatory motif.

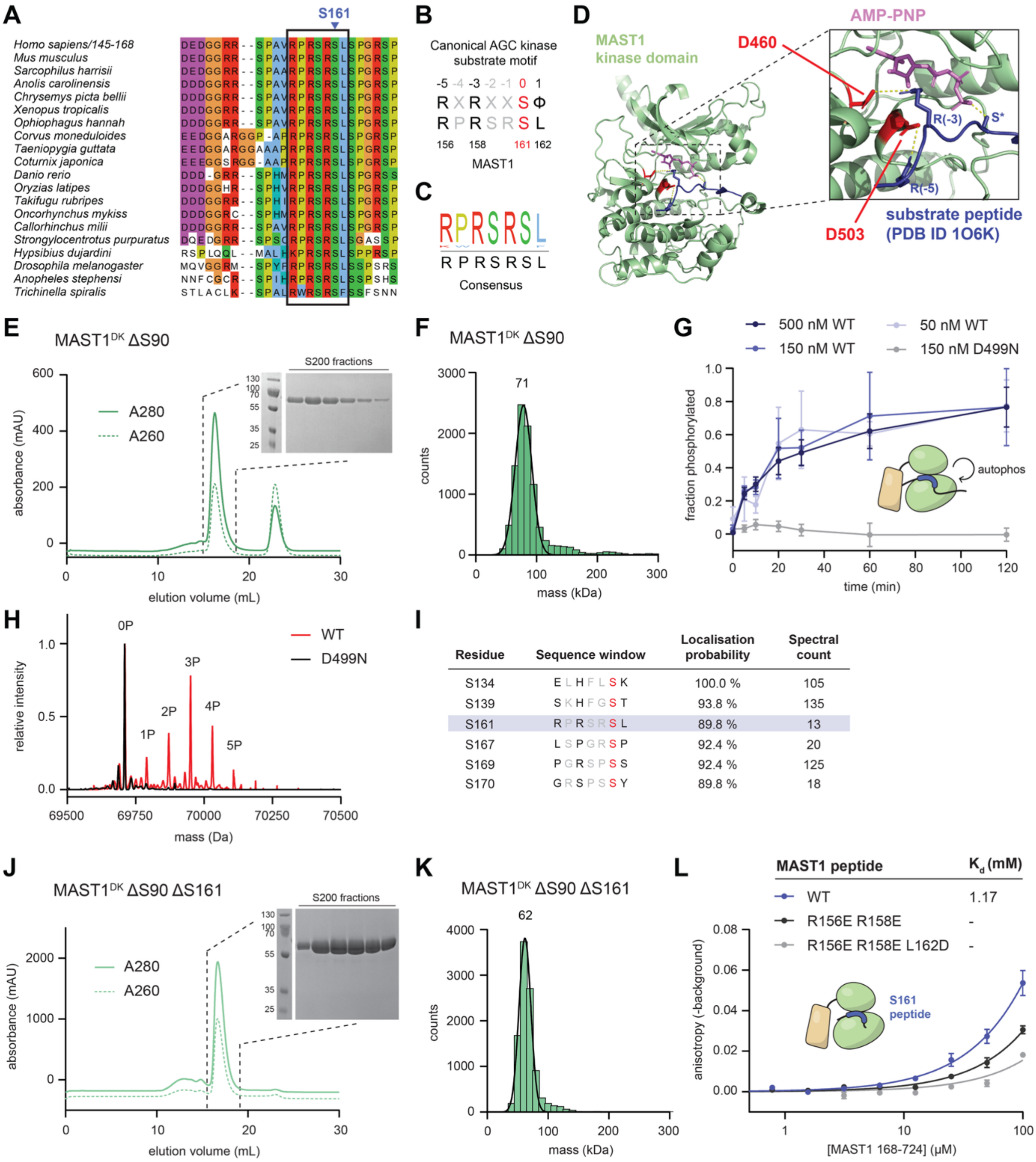

A. Multiple sequence alignment of MAST1 residues surrounding S161 from *Nematoda* to *Mammalia*. S161 residue highlighted above, AGC kinase recognition region outlined in black box.

B. Alignment of canonical AGC kinase substrate motif with $MAST1^{156-162}$, where S161 is the phosphorylatable residue (red) and R156 (-5), R158 (-3), and L162 (+1) are in positions specifically involved in substrate recognition (black).

C. Consensus sequence of $MAST1^{156-162}$ from sequence alignment in panel A.

D. MAST1 kinase domain (green) with GSK3 substrate peptide (dark blue, S* is phospho-acceptor) from superimposed crystal structure of AKT2 (PDB ID: 1O6K) (7). MAST1 possesses two conserved aspartate residues (D460 and D503, mouse numbering, red) in position to bind the P-3 and P-5 arginines of a canonical AGC kinase recognition motif.

E. Size exclusion chromatography (SEC) elution profile of purified $MAST1^{DK}$ ΔS90. Coomassie-stained SDS-PAGE gel of SEC fractions spanning elution peak shown in inset.

F. Mass photometry of purified $MAST1^{DK}$ ΔS90. Theoretical mass = 70 kDa, observed mass = 71 kDa.

G. Radiometric autophosphorylation assay with various concentrations of $MAST1^{DK}$ ΔS90 WT or kinase dead (D499N). Data are presented as mean values ± S.E., n = 3.

H. Intact MS analysis of $MAST1^{DK}$ ΔS90 WT (red) and kinase dead (D499N) (black) after autophosphorylation shown in panel G. Intensity of both samples were normalized to a scale of 0 to 1. Addition of phosphate in WT sample (1P, 2P etc.) detected by each 80 Da shift.

I. Tandem MS analysis of $MAST1^{DK}$ ΔS90 WT after autophosphorylation shown in panel G. Phosphorylated serines are indicated in red and residues involved in AGC kinase binding (-5, -3, +1) are in black. Top six serine sites (by spectral count) shown in table, S161 highlighted in dark blue. See Supplemental File 3 for full data.

J. SEC elution profile of purified $MAST1^{DK}$ ΔS90 ΔS161. Coomassie-stained SDS-PAGE gel of SEC fractions spanning elution peak shown in inset.

K. Mass photometry of purified $MAST1^{DK}$ ΔS90 ΔS161. Theoretical mass = 64 kDa, observed mass = 62 kDa.

L. Binding curves for $MAST1^{DK}$ ΔS90 ΔS161 and MAST1 peptides spanning S161, measured by fluorescence anisotropy. Data are presented as mean values ± S.E., *n* = 3. Data were fit with a one-site binding model and the K_d derived from curve fitting.

Figure S8. Reconstitution of MAST1:14-3-3η complexes *in vitro*.

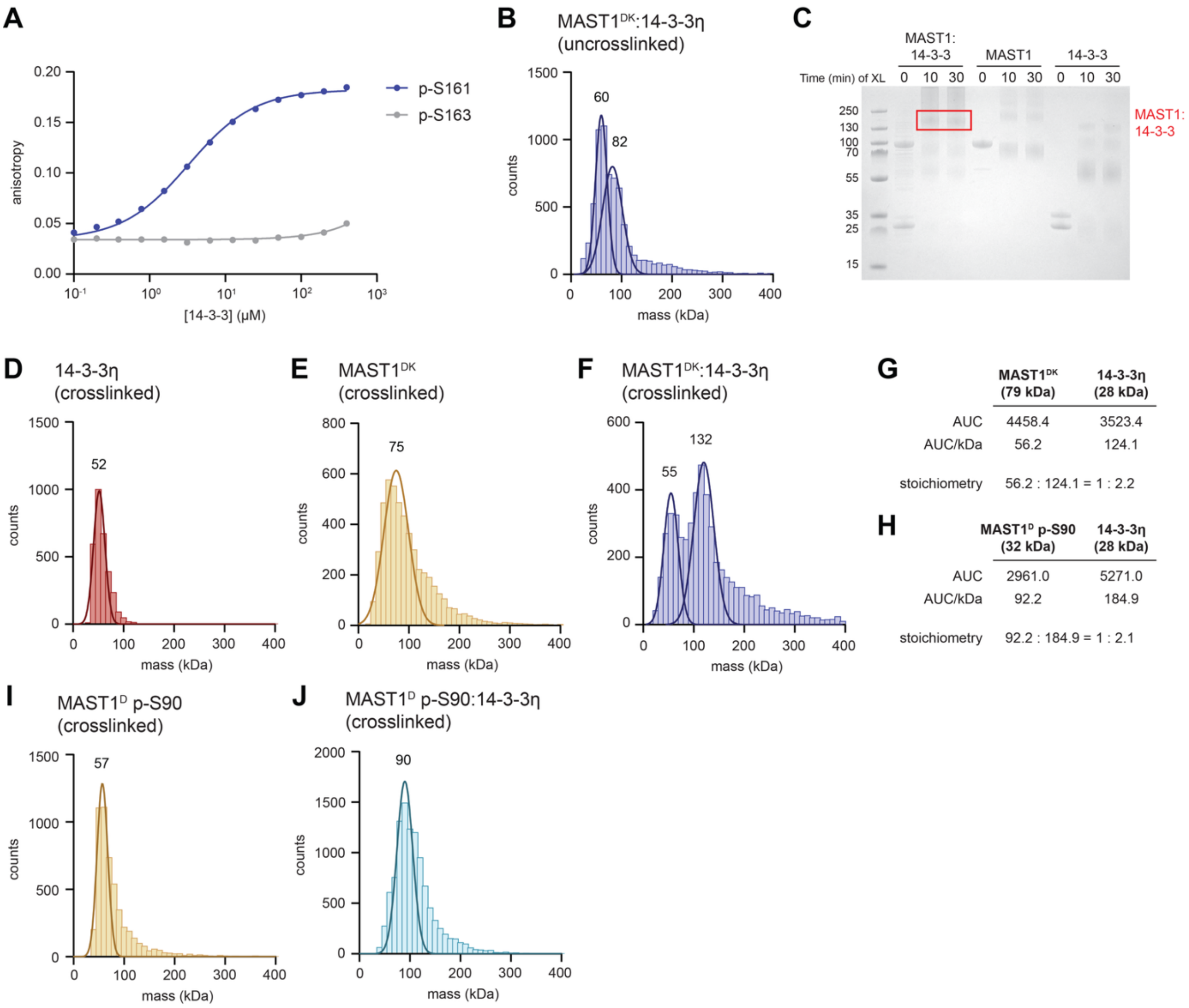

A. Binding curves for 14-3-3η and MAST1 peptides spanning S161 or S163, measured by fluorescence anisotropy. Data are presented as mean values ± S.E., *n* = 3. Data were fit with a one-site binding model and the K_d derived from curve fitting.

B. Mass photometry of co-purified $MAST1^{DK}$ and 14-3-3η complex.

C. Coomassie-stained SDS-PAGE gel of $MAST1^{DK}$ and 14-3-3η complex, $MAST1^{DK}$ and 14-3-3η after 0, 10, and 30 min of glutaraldehyde crosslinking. Crosslinked trimeric complex outlined in red box.

D. Mass photometry of purified 14-3-3η after 10 min of glutaraldehyde crosslinking.

E. Mass photometry of purified MAST1^{DK} after 10 min of glutaraldehyde crosslinking.

F. Mass photometry of co-purified MAST1^{DK} and 14-3-3η complex after 10 min of glutaraldehyde crosslinking.

G. Densitometry analysis of Coomassie-stained, copurified MAST1^{DK} and 14-3-3η in the second lane of the gel in Figure 3B. Band intensity was measured as area under the curve (AUC) in ImageJ, then normalized by the molecular weight of the respective protein.

H. Densitometry analysis of Coomassie-stained, copurified MAST1^{D} p-S90 and 14-3-3η in the third lane of the gel in Figure 3F. Band intensity was measured as area under the curve (AUC) in ImageJ, then normalized by the molecular weight of the respective protein.

I. Mass photometry of purified MAST1^{D} p-S90.

J. Mass photometry of *in vitro*-formed MAST1^{D} p-S90 and 14-3-3η complex after 10 min of glutaraldehyde crosslinking.

Figure S9. Tau is a candidate MAST1 substrate.

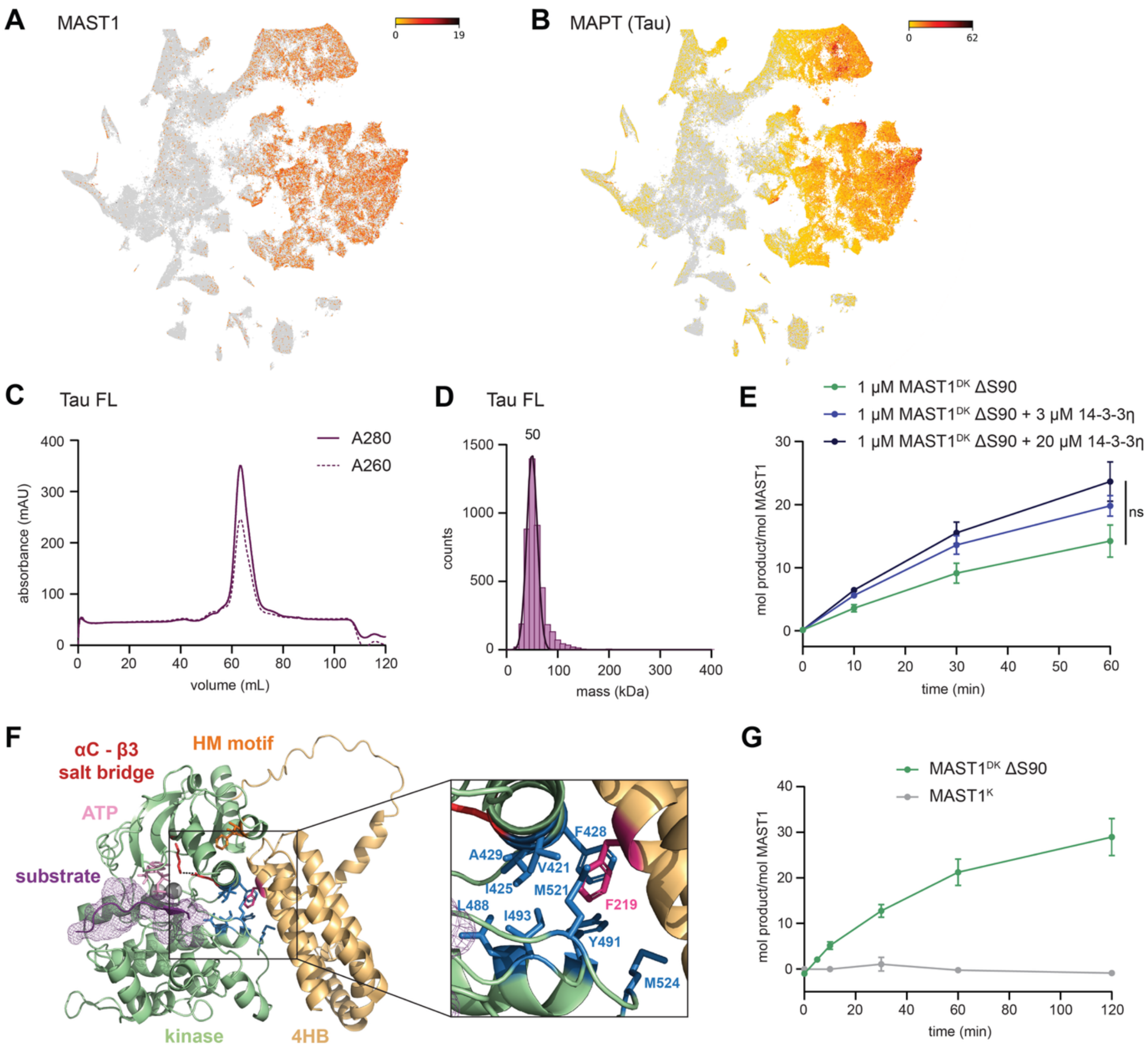

A. MAST1 expression levels overlayed on t-SNE plot from Figure S2A.

B. MAPT (Tau) expression levels overlayed on t-SNE plot from Figure S2A.

C. SEC elution profile of purified Tau.

D. Mass photometry of purified Tau. Theoretical mass = 42 kDa, observed mass = 50 kDa.

E. Radiometric kinase assay of Tau phosphorylation by MAST1^{DK}, MAST1^{DK} and 14-3-3η complex, or MAST1^{DK} and 14-3-3η complex supplemented with excess of purified 14-3-3η (n = 3, p = 0.0642, two-way repeated-measures ANOVA). Data are presented as mean values ± S.E.

F. Alphafold 3 prediction of MAST1 kinase domain (green) and 4HB (yellow) with GSK3 substrate peptide (purple mesh), ATP analog (pink) and Mg^{2+} ions (grey) from superimposed crystal structure of AKT2 (PDB ID: 1O6K) (7). The kinase domain is in the active conformation, evident by the presence of a salt bridge between the αC helix and the β3 strand (K403-E422, red), and the docking of the hydrophobic motif (HM) into the hydrophobic pocket of the N-lobe (I703 and F706, orange). The hydrophobic residues involved in the interface between the kinase domain and the 4HB are highlighted in blue and pink, respectively.

G. Radiometric kinase assay by $MAST1^{DK}$ ΔS90 or $MAST1^{K}$ on Tau. Data are presented as mean values ± S.E., n = 3.

Figure S10. MCC-CH-CM mutations perturb MAST1 folding and kinase activity.

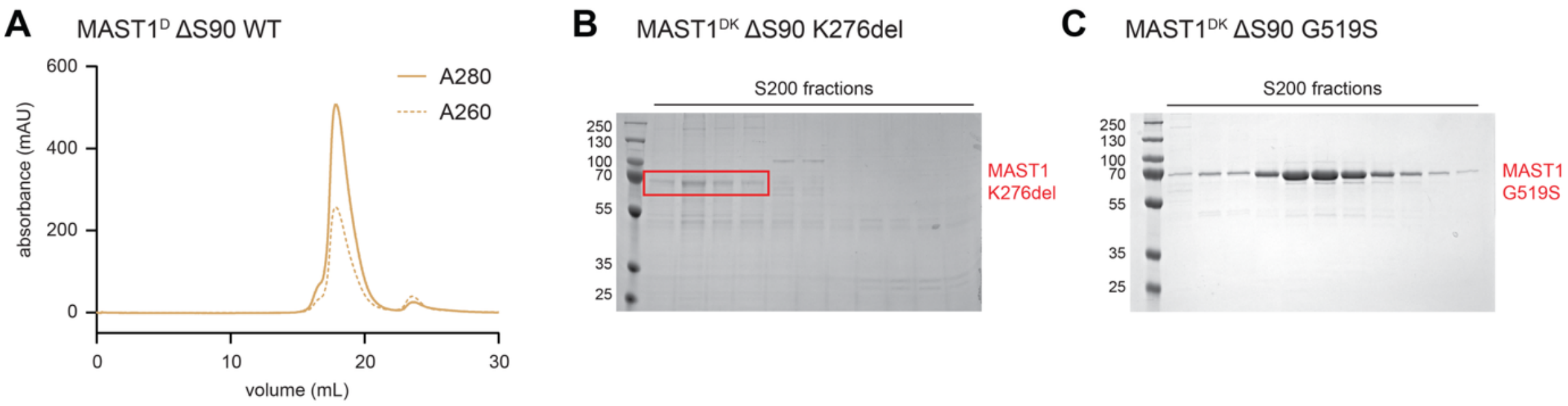

A. SEC elution profile of purified MAST1^{D} ΔS90 WT.

B. Coomassie-stained SDS-PAGE gel of SEC fractions spanning elution peak of MAST1^{DK} ΔS90 K276del. Bands representing MAST1 outlined in red box.

C. Coomassie-stained SDS-PAGE gel of SEC fractions spanning elution peak of MAST1^{DK} ΔS90 G519S.

Supplementary Tables

Supplementary Table 1. Oligonucleotide sequences for generation of G519S mouse model

mMAST1-G517S sgRNA target sequence	CACATCAAGCTCACGGATTTTGG
mMAST1-G517S ssDNA repair template	CCTGAGATCCCCACCTGTGCCTACAGCCTCCTTATCACCTCCATGGGTCACATCAAGCTCACAGATTTCGGCCTCTCCAAGATGGGGCTCATGAGCCTCACCACCAACTTATATGAAGGCCACATCGAGAAGGACGCCCC
mMAST1-G517S genotyping primer fw	CTCAAGCCAGACAAGTGAG
mMAST1-G517S genotyping primer rv	CCTGTTTGTCCAGGAACTC

Supplementary Files 1-4 Legends

Supplementary File 1. MAST1 truncations AP-MS

Complete MS2 dataset from EGFP-MAST1 AP-MS experiment, comparing EGFP, EGFP-MAST1^{DKP}, EGFP-MAST1^{DKP} ΔS90, and EGFP-MAST1^{DKP} ΔS90 ΔS161.

Supplementary File 2. MAST1 mutants AP-MS

Complete MS2 dataset from EGFP-MAST1 AP-MS experiment, comparing EGFP-MAST1^{DKP} WT, EGFP-MAST1^{DKP} S90A, EGFP-MAST1^{DKP} S161A, and EGFP-MAST1^{DKP} S90A S161A.

Supplementary File 3. MAST1 mutant AP-MS with dorsal brain organoid lysates

Complete MS2 dataset from EGFP-MAST1 AP-MS experiment using lysates of day 120 dorsal brain organoids, comparing EGFP, EGFP-MAST1^{DKP} WT and EGFP-MAST1^{DKP} S90A S161A.

Supplementary File 4. All phosphomapping MS2 experiments

Complete MS2 datasets of all phosphomapped proteins, including PAK4-phosphorylated MAST1^{D} (Figure 2E), autophosphorylated MAST1^{DK} ΔS90 (Figure S7I), MAST1^{DK}:14-3-3η complex co-expressed and purified from Sf9 insect cells (Figure 3C), and MAST1-phosphorylated Tau (Figure 5H).

Supplementary File 5. Phosphoproteomics L278del G519S overlap

Full dataset of phosphoproteomics experiment comparing MAST1 WT with L278del and G519S.

Supplementary Methods

Protein expression and purification

Sf9 expression

All proteins expressed are derived from *Mus musculus* sequence unless otherwise stated. TEV-cleavable N-terminal 10xHis-tagged constructs of MAST1^{DK}, MAST1^{DK} ΔS90, MAST1^{DK} ΔS90 ΔS161, and PAK4^{FL}, 3C-cleavable N-terminal GST-tagged constructs of 14-3-3η, and a TEV-cleavable N-terminal StrepII-tagged construct of *Homo sapeins* MRCKα^{FL} were cloned into the pFastBacDual vector and expressed in baculovirus-infected *Spodoptera frugiperda* (Sf9) insect cells. Mutant constructs were cloned using site-directed mutagenesis. Sf9 cell pellets from 1 L of expression culture were lysed in 100 mL lysis buffer (50 mM HEPES pH 7.4, 150 mM NaCl, 1 mM TCEP, 2 mM benzamidine, 2 mM $MgCl_2$, 1x protease inhibitor cocktail (Sigma P8849), 1 kU Denarase (c-LEcta)). 20 mM imidazole was added for constructs purified by Ni-NTA affinity chromatography, 0.25% CHAPS was added for constructs purified by GST-beads, and phosphatase inhibitors (50 mM NaF, 10 mM betaglycerophosphate, 2 mM sodium pyrophosphate, 1 mM sodium orthovanadate) was added for MAST1^{DK}/14-3-3η co-purification to retain phosphates. Cleared lysates were obtained by centrifugation (38,000 g, 4°C, 30 min).

BL21 expression

All proteins expressed are derived from *Mus musculus* sequence unless otherwise stated. TEV-cleavable N-terminal 6xHis-tagged constructs of MAST1^{D}, 14-3-3η, and 14-3-3θ were cloned into the pHisII vector, an N-terminal 6xHis-tagged construct of Tau^{FL} (isoform F) was cloned into the pET28b vector, and a TEV-cleavable N-terminal GST-tagged construct of *Homo sapiens* MLC2^{FL} was cloned into the pGST2 vector, for expression in BL21 Star *E. coli*. The cells were grown at 37°C to an OD of 0.7 before induction with 250 µM IPTG and overnight expression at 18°C. BL21 cell pellets from 1 L of expression culture were lysed in 50 mL lysis buffer (50 mM HEPES pH 7.4, 150 mM NaCl, 1 mM TCEP, 2 mM benzamidine, 2 mM $MgCl_2$, 1x protease inhibitor cocktail (Sigma P8849), 0.5 kU Denarase (c-LEcta), 5 mg lysozyme). 20 mM imidazole and 0.25% CHAPS

was added as described above. Lysates were sonicated three times for 3 min at 50% amplitude and cleared lysates were obtained by centrifugation (38,000 g, 4°C, 20 min).

Purification

For Ni-NTA affinity purification, cleared lysates were run over a HisTrap FF column (Cytiva), washed with buffer A (50 mM HEPES pH 7.4, 150 mM NaCl, 1 mM TCEP) followed by buffer A with 2% buffer B (buffer A + 1 M imidazole), and eluted with buffer A with 30% buffer B. The eluate was cleaved and dialyzed overnight at 4°C with 8.8 nmol TEV protease (purified in house) in 50 mM HEPES pH 7.4, 50 mM NaCl, and 1 mM TCEP. Constructs requiring dephosphorylation were treated with 2.3 nmol of lambda-phosphatase (purified in house) and 0.1 mM $MnCl_2$.

For GST affinity purification, cleared lysates were incubated with 3 mL glutathione sepharose beads (Cytiva) for 3 hours at 4°C. The beads were washed three times with buffer A (50 mM HEPES pH 7.4, 150 mM NaCl, 1 mM TCEP, 0.25% CHAPS) and the construct was cleaved off the beads overnight at 4°C with either 8.8 nmol TEV protease (purified in house) or 11.1 nmol 3C protease (purified in house).

For StrepII affinity purification, cleared lysates were run over a StrepTrap HP column (Cytiva), washed with buffer A (50 mM HEPES pH 7.4, 150 mM NaCl, 1 mM TCEP), and eluted with buffer B (buffer A + 2.5 mM d-desthiobiotin). The eluate was cleaved overnight at 4°C with 8.8 nmol TEV protease (purified in house).

Following affinity purification, constructs underwent ion-exchange purification. Proteins that were not dialyzed to 50 mM NaCl were first diluted to make a final concentration of 50 mM NaCl, then run over either a HiTrap Q HP or HiTrap SP HP column (Cytiva), washed with buffer A (50 mM HEPES pH 7.4, 1 mM TCEP), and eluted with a gradient of buffer B (50 mM HEPES pH 7.4, 1 M NaCl, 1 mM TCEP). The peak fractions were pooled, concentrated, and injected onto a size exclusion column (Cytiva) equilibrated in 50 mM HEPES pH 7.4, 150 mM NaCl, 1 mM TCEP. S200 and S75 columns were used for constructs >30 kDa and <30 kDa respectively, and 10/300 and 16/600 columns were used for yields >5 mg and <5 mg respectively. For $MAST1^{D}$ requiring phosphorylation on S90, it was incubated with purified $PAK4^{FL}$ at room temperature for 2 hours prior to injection on the size exclusion column. The peak fractions were pooled, concentrated, and stored at -80°C.

Cell culture

HEK293 cells were maintained in DMEM supplemented with 10% FBS. Cells were tested negative for *Mycoplasma* contamination using Eurofins Genomics Mycoplasmacheck service.

Preparation of dorsal brain organoid lysates

Stem Cell Culture

Feeder-free WA09 (H9) hESCs (WiCell) were maintained on Matrigel-coated plates (Corning) in mTeSR (Stem Cell Technologies) at 37 °C and 5% CO_2. For passaging, colonies were detached by incubating in 0.5 mM EDTA in DPBS-/- (Gibco) for 5 min at 37 °C before replating in mTeSR medium.

Cerebral organoid generation

Cerebral organoids were generated as previously described (8). Briefly, a single cell suspension of H9 hESCs was made by incubation for 5 min with Accutase (Merck). On day 0, 9000 cells were seeded in a 96-well ultra-low-attachment U-bottom plate (Sigma) in 150 µl of mTeSR supplemented with RevitaCell (Thermo Fisher Scientific) to form embryoid bodies. Media was changed to mTeSR on day 3, and to neural induction medium on day 6. Organoids were embedded in Matrigel (Corning) on day 12, and 3 µM of CHIR99021 (Merck) was added to the neural induction medium on day 13-16. On day 16, media was switched to improved differentiation medium supplemented with B27 minus vitamin A (IDM), and on day 25 to improved differentiation medium supplemented with B27 plus vitamin A (IDM+A). Media was replenished every 3-4 days and organoids were grown for a total of 120 days, then snap-frozen (without media) in liquid nitrogen and stored at -80°C.

Lysis

Eight organoids per replicate were thawed in 1.25 mL of lysis buffer (10 mM monosodium phosphate, 40 mM disodium phosphate, 10 mM NaCl, 1 mM TCEP, 2 mM benzamidine, 2 mM $MgCl_2$, 1x protease inhibitor cocktail (Sigma P8849), 1 kU Denarase (c-LEcta), 0.25 CHAPS, 50 mM NaF, 10 mM beta-glycerophosphate, 2 mM sodium pyrophosphate, 1 mM sodium orthovanadate) by incubating with rotation at 4°C for 30 min. The lysate was passed three times through a 21G syringe then spun at 7,000 g at 4°C for 15 min. The supernatant was split equally between all samples within one replicate and used for incubation with EGFP-MAST1^{DKP} immobilized on GFP-Trap

Magnetic beads (Chromotek), with rotation at 4°C for 2 h. Preparation of EGFP-MAST1 on beads, as well as all downstream steps, are described in EGFP-MAST1 AP-MS methods in the main text.

Intact mass spectrometry

Protein sample was diluted in dH2O to 4 ng/µL and dithiothreitol was added to a final concentration of 25 mM. 8 ng (2 µL) of protein was loaded on an XBridge Protein BEH C4 column (2.5 µm particle size, dimensions 2.1 mm x 150 mm; Waters) using a Vanquish™ Horizon UHPLC system (Thermo Scientific) with a working temperature of 50 °C, 0.1% formic acid (FA) as solvent A, 100% acetonitrile, 0.08% FA as solvent B. Proteins were separated on a 6 min step gradient from 12 to 72% solvent B at a flow rate of 250 µL/min and analyzed on a Synapt G2-Si coupled via a ZSpray ESI source (Waters). Data were recorded with MassLynx V 4.2 (Waters) and analyzed using the MaxEnt 1 process to reconstruct the uncharged average protein mass.

Tandem mass spectrometry

Sample preparation

For MAST1^{D}, MAST1^{DK} ΔS90, and MAST1^{DK}:14-3-3η, ~5 µg protein in 1.5 µL was diluted with 8.5 µL of 8 M urea, 50 mM ammonium bicarbonate (ABC). For Tau^{FL}, ~5 µg of protein in 2.5 µL was mixed with 10% sodium deoxycholate (SDC) and 50 mM ABC to reach 2% SDC. Proteins were reduced with 0.4 µL of 250 mM dithiothreitol (DTT) for 30 min at room temperature (50°C for Tau^{FL}) and alkylated by adding 0.4 µL of 500 mM IAA and incubating for 30 min at room temperature in the dark. The remaining iodoacetamide (IAA) was quenched by adding 0.2 µL of 250 mM DTT for 10 min. For MAST1^{D}, MAST1^{DK} ΔS90, and MAST1^{DK}:14-3-3η, the sample was diluted to below 1 M urea with 70 µL of 50 mM ABC. For Tau^{FL}, the sample was diluted to 1% SDC with 10 µL of 100 mM ABC. Proteins were digested by adding ~160 ng trypsin (Trypsin Gold, Promega) and incubated at 37°C overnight. The digest was stopped by the addition of 10% trifluoroacetic acid to a final concentration of 0.5% (1% for Tau^{FL}). For Tau^{FL}, samples were centrifuged at 18,500 x g for 10 min to remove the SDC, the supernatant was transferred to a new tube, 10% TFA was added to reach a final concentration of 2%, and the sample was centrifuged again at 18,500 x g for 10 min. The resulting supernatant was desalted using C18 Stagetips (9).

For EGFP-MAST1 AP-MS bead samples, the beads were resuspended in 30 µL of 2 M urea and 50 mM ammonium bicarbonate. Disulfide bonds were reduced with 1.2 µL of 250 mM DTT for 30 min at room temperature before adding 1.2 µL of 500 mM IAA and incubating for 30 min at room temperature in the dark. The remaining iodoacetamide was quenched with 0.6 µL of 250 mM DTT for 10 min. Proteins were digested with 150 ng LysC (mass spectrometry grade, FUJIFILM Wako chemicals) and trypsin (Trypsin Gold, Promega) mix at room temperature for 90 min. The supernatant was transferred to a new tube and the beads were rinsed with 30 µL of 2 M urea and 50 mM ammonium bicarbonate. 60 µL of 50 mM ammonium bicarbonate was added to the tube, before digestion with 150 ng LysC/trypsin mix at 37°C for 5 h. The digest was stopped by the addition of TFA to a final concentration of 0.5%, and the peptides were desalted using C18 Stagetips (9).

Liquid chromatography-mass spectrometry analysis

For MAST1^{D}, MAST1^{DK} ΔS90, MAST1^{DK}:14-3-3η, and Tau^{FL}, LC-MS analysis was performed on a Vanquish Neo UHPLC system (Thermo Scientific) coupled to an Orbitrap Exploris 480 mass spectrometer (Thermo Scientific). The system was equipped with a Nanospray Flex ion source (Thermo Scientific), coated emitter tips (PepSep, MSWil), and a Butterfly Portfolio Heater (Phoenix S&T). Peptides were loaded onto a trap column (Acclaim PepMap 100 C18 HPLC Column, 20 mm × 0.1 mm, 5 µm particle size, Thermo Scientific) using 0.1% TFA as mobile phase, and separated on an analytical column (Acclaim PepMap 100 C18 HPLC Column, 50 cm × 75 µm, 2 µm particle size, Thermo Scientific), applying a linear gradient starting with a mobile phase of 98% solvent A (0.1% FA) and 2% solvent B (80% acetonitrile, 0.08% FA), increasing to 35% solvent B over 60 min at a flow rate of 230 nL/min. The analytical column was heated to 30°C.

The mass spectrometer was operated in data-dependent acquisition (DDA) mode, with 2 s MS1 cycle time. For MAST1^{D}, MAST1^{DK} ΔS90, and MAST1^{DK}:14-3-3η, survey scans were acquired from 375-2000 m/z with lock mass enabled, normalized AGC target of 300%, resolution of 120,000. For Tau^{FL}, survey scans were acquired from 340-1500 m/z with lock mass enabled, normalized AGC target of 300%, resolution of 60,000. The most intense precursor ions (charge states +2 to +6) were selected for fragmentation using an isolation window of 1.2-1.4 m/z. Selected ions were analyzed with a maximum fill time of 100 ms, normalized AGC target of 200%, and resolution of 30,000 after HCD

fragmentation with normalized collision energy of 28-30%. Monoisotopic precursor selection (MIPS) was set to "peptide" mode, the intensity threshold to 2.5 x 10^4 (1 x 10^4 for Tau^{FL}), and selected precursors were dynamically excluded for 20 seconds with isotope exclusion enabled.

For EGFP-MAST1 AP-MS samples, LC-MS analysis was performed on an UltiMate 3000 RSLCnano LC system (Thermo Scientific) coupled to a timsTOF HT (Bruker). The system was equipped with a CaptiveSpray ion source (Bruker), and a Butterfly Portfolio Heater (Phoenix S&T). Peptides were loaded onto a trap column (Acclaim PepMap 100 C18 HPLC Column, 20 mm × 0.1 mm, 5 µm particle size, Thermo Scientific) using 0.1% TFA as mobile phase, and separated on an analytical column (Aurora Ultimate XT C18, 25 cm × 75 µm, 1.7 µm particle size, IonOpticks), applying a linear gradient starting with a mobile phase of 98% solvent A (0.1% FA) and 2% solvent B (80% acetonitrile, 0.08% FA), increasing to 35% solvent B over 60 min at a flow rate of 300 nL/min. The analytical column was heated to 50°C.

The mass spectrometer was operated in data-independent acquisition (DIA) parallel accumulation serial fragmentation (PASEF) mode. MS2 data were acquired with eight PASEF scans per duty cycle, each containing three m/z windows. The m/z window widths were adjusted based on the expected precursor density, covering a total range of 300–1200 m/z. The ion mobility range was set to 0.64-1.42 V*s/cm, and the accumulation and ramp time was set to 100 ms. TIMS elution voltages were calibrated linearly to obtain the reduced ion mobility coefficients (1/K0) using three Agilent ESI-L Tuning Mix ions (m/z 622, 922 and 1,222). Collision energy for fragmentation was scaled linearly with precursor mobility (1/K0), ranging from 20 eV (at 1/K0 = 0.6 V*s/cm) to 59 eV at (at 1/K0 = 1.6 V*s/cm).

Mass spectrometry data analysis

For $MAST1^{D}$, $MAST1^{DK}$ ΔS90, $MAST1^{DK}$:14-3-3η, and Tau^{FL}, MS raw data were analyzed with FragPipe (22.0), using MSFragger (4.1) (10), IonQuant (1.10.27) (11), and Philosopher (5.1.1) (12). The default FragPipe workflow for label free quantification (LFQ-MBR) was used, except "Normalize intensity across runs" was turned off. Cleavage specificity was set to Trypsin/P, with two missed cleavages allowed. The protein FDR was set to 1%. Carbamidomethyl was used as fixed cysteine modification; methionine oxidation, phosphorylation at STY and protein N-terminal acetylation were specified as

variable modifications. PTMProphet was used for the PTM site localization. MS2 spectra were searched against the *Spodoptera frugiperda* protein sequences from Uniprot (Taxon ID: 7108, release 2024_01), concatenated with a database of 379 common laboratory contaminants (release 2023.03, https://github.com/maxperutzlabs-ms/perutz-ms-contaminants), and protein sequences for the constructs.

For EGFP-MAST1 AP-MS samples, MS raw data were processed with Spectronaut (19.0 or 19.2, Biognosys). The library-free DirectDIA+ workflow was employed for analysis of the raw files, the *H. sapiens* 1 protein per gene reference proteome from Uniprot (Proteome ID: UP000005640, release 2024_01), concatenated with a database of 379 common laboratory contaminants (release 2023.03, https://github.com/maxperutzlabs-ms/perutz-ms-contaminants), and sequences for the constructs. The cleavage specificity was set to full trypsin specificity (Trypsin/P), with two missed cleavages allowed. Carbamidomethyl was used as fixed cysteine modification; methionine oxidation and protein N-terminal acetylation were specified as variable modifications. Cross-run normalization was disabled, and all other settings were used at their default values.

Post-processing with in-house pipeline

Computational analysis was performed using Python and the in-house developed Python library MsReport (0.0.26) (13). Only non-contaminant proteins identified with a minimum of two peptides and being quantified once were considered for further analysis. LFQ protein intensities were log2-transformed and normalized across samples using the ModeNormalizer from MsReport. The ModeNormalizer method involves calculating log2 protein ratios for all pairs of samples and determining normalization factors based on the modes of all ratio distributions. Missing values were imputed by drawing random values from a normal distribution. Sigma and mu of this distribution were calculated per sample from the standard deviation and median of the observed log2 protein intensities (μ = median sample LFQ intensity – 1.8 standard deviations of the sample LFQ intensities, $\sigma = 0.3 \times$ standard deviation of the sample LFQ intensities). iBAQ intensities were calculated by dividing protein intensities by the number of theoretically observable tryptic peptides between 6 and 30 amino acids. To estimate the relative protein abundances within each sample, the iBAQ intensities were normalized by dividing them by the total sum of iBAQ intensities for all proteins in the sample. For

the EGFP-MAST1 AP-MS samples, statistical analysis was performed using the linear models for microarray analysis (limma) v.3.54.2 (14) package in R. Moderated t-statistics were calculated using the limma-trend method, and multiple testing correction was applied using the Benjamini–Hochberg method. The Python library XlsxReport (0.1.1) (15) was used to create formatted Excel files summarizing the results of the proteomics experiments.

Data availability

The mass spectrometry proteomics data have been deposited to the ProteomeXchange Consortium via the PRIDE (16) partner repository with the data set identifiers PXD063727, PXD063803, PXD063802, PXD063795, PXD063731 and PXD080824.

Phosphoproteomics

Methanol/Chloroform precipitation and proteolytic digest

Cortical P0 samples were extracted from n = 5 WT and homozygous litter-matched P0 animals of both the MAST1 L278del and the MAST1 G519S mouse line and flash-frozen for storage. The cortices were dissolved in 500 µL methanol and then protein was precipitated by adding 1 mL chloroform. After centrifugation (10 min, 10,000 g) the supernatant was removed. The pellets were lysed in a solution of 10 M urea and 50 mM HCl and then 1 M Triethylammoniumbicarbonate (TEAB) was added to a final concentration of 100 mM.

The lysates were supplemented with 5 mM DTT and incubated at 37°C for 1 h. The alkylation was performed by adding 10 mM Iodoacetamide and incubating for 30 min in the dark. The reaction was quenched by addition of 7.5 mM DTT and incubation at room temperature for 30 min. The samples were diluted to 6 M urea by addition of 100 mM TEAB and the proteins were digested with lysyl endopeptidase (Lys-C, Fujifilm Wako Pure Chemical Corporation) at an enzyme to protein ration of 1:50 for 3 h at 37°C. Subsequently, the samples were further diluted with 100 mM TEAB Buffer to 2 M urea, and trypsin (Trypsin Gold, Promega) was added at an enzyme to protein ratio of 1:50 and the samples were incubated overnight at 37°C.

The samples were acidified to a pH below 2 with 10% TFA and were desalted using C18 cartridges (Sep-Pak Vac 1cc (200 mg), Waters). Peptides were eluted with 2 x 600 µL 80% Acetonitrile (ACN) and 0.1% Formic Acid (FA), followed by freeze-drying.

TMT labelling

1.5 mg of lyophilized peptides were dissolved in 240 µL of 100 mM Triethylammonium bicarbonate. TMT10-plex labelling reagents (3 vials per label) were dissolved in 41 µL of anhydrous acetonitrile. 3 vials per label (2.4 mg of reagent) were added to the peptide samples and incubated for 1 h. After quenching with 5% hydroxylamine, the samples were mixed in several steps to achieve a correct mixing ratio of 1:1:1:1:1:1:1:1:1:1. (median of Top 500 proteins)

Following labelling, peptides were again freeze-dried, dissolved in TFA 0.1% and split in two aliquots: one containing 500 µg for measurement of the unmodified peptides and proteome quantification and one containing 13 mg for enrichment of phosphopeptides and phosphoproteome quantification. Both were desalted using C18 cartridges (Sep-Pak, Waters). Peptides were eluted with 70% ACN and 0.1% FA, followed by freeze-drying.

Phosphopeptide enrichment

Phosphopeptides were enriched using TiO_2 by first dissolving the dried peptide mixture in 4 mL of TiO_2 loading buffer (300 mg/mL lactic acid (Sigma-Aldrich), 80% ACN, 0.1% TFA) and incubating them with 120 mg of TiO_2 resin (Titansphere bulk media, 5 µm, GL Science) for 60 min at room temperature with over end rotation. For washing and elution the bead suspension was split and transferred to 8 Mobicol (MoBiTec) spin columns with a 10 µm pore filter at the outlet.

The TiO_2 beads in each spin column were washed with 8 x 500 µL of TiO_2 loading buffer, 8 x 500 µL of 80% ACN, 0.1% TFA, 4 x 500 µL of 1% ACN, 0.1% TFA, 4 x 500 µL of 1% ACN, 0.1% FA. Bound peptides were eluted from the resin by addition of 3 x 200 µL 0.3 M NH_4OH, 2 x 200 µL 0.7 M NH_4OH and 2 x 150 µL 0.4 M NH_4OH and 40% ACN, eluates were unified and freeze-dried.

Fractionation of total peptides and enriched phosphopeptides by SCX

The lyophilized peptide samples were dissolved in SCX buffer A (5 mM phosphate buffer pH 2.7, 15% ACN). 200 µg of the unmodified peptides and the total amount of the

enriched phosphopeptides were loaded on the SCX column. The SCX fractionation was performed on an Ultimate system (Thermo Fisher Scientific) using a TSKgel SP-25W (ToSOH) column (5 µm particles, 1 mm i.d. x 300 mm) at a flow rate of 40 µL/min.

For the separation a ternary gradient was used, starting with 100% buffer A for 10 min, followed by a linear increase to 10% buffer B (5 mM phosphate buffer pH 2.7, 1 M NaCl, 15% ACN) and 50% buffer C (5 mM phosphate buffer pH 6.0, 15% ACN) in 15 min (for proteome) or 30 min (for phosphoproteome), to 25% buffer B and 50% buffer C in next 5 min (for proteome) or 10 min (for phosphoproteome), to 50% buffer B and 50% buffer C in next 5 min and an isocratic elution for further 15 min.

The flow-through was collected as a single fraction, along the gradient fractions were collected every minute and stored before analysis.

LC-MS/MS

The nano HPLC system used was an UltiMate 3000 HPLC RSLC nano system (Thermo Scientific) coupled to a Q Exactive HF-X mass spectrometer (Thermo Scientific), equipped with a Proxeon nanospray source (Thermo Scientific). Peptides were loaded onto a trap column (Thermo Scientific, PepMap C18, 5 mm × 300 µm ID, 5 µm particles, 100 Å pore size) at a flow rate of 25 µL/min using 0.1% TFA as mobile phase. After 10 min, the trap column was switched in line with the analytical column (Thermo Scientific, PepMap C18, 500 mm × 75 µm ID, 2 µm, 100 Å). Peptides were eluted using a flow rate of 230 nL/min and a binary 120 min gradient. The two-step gradient started with the mobile phases: 98% A (water/formic acid, 99.9/0.1, v/v) and 2% B (water/acetonitrile/formic acid, 19.92/80/0.08, v/v/v) increased to 40% B over the next 120 min, followed by a gradient in 5 min to 95% B, stayed there for 5 min and decreased in 2 min back to 98% A and 2% B for equilibration at 30°C.

The Q Exactive HF-X mass spectrometer was operated in data-dependent mode, using a full scan (m/z range 380-1650, nominal resolution of 120,000, target value 3E6) followed by MS/MS scans of the 10 most abundant ions. MS/MS spectra were acquired using normalized collision energy of 35, isolation width of 0.7 m/z, resolution of 45,000, a target value of 1E5 and maximum fill time of 250 ms. For the detection of the TMT reporter ions a fixed first mass of 110 m/z was set for the MS/MS scans. Precursor ions selected for fragmentation (exclude charge state 1, 7, 8, >8) were put on a dynamic

exclusion list for 30 s. Additionally, the minimum AGC target was set to 1E4 and intensity threshold was 4E4. The peptide match feature was set to preferred and the exclude isotopes feature was enabled.

Data processing

For peptide identification, the RAW-files were loaded into Proteome Discoverer (version 2.3.0.523, Thermo Scientific). All hereby created MS/MS spectra were searched using MSAmanda v2.3.0.12368, engine version 2.0.0.12368 (17). The RAW-files were searched against the Uniprot reference database, using mouse as sub-organism (21,963 sequences; 11,719,000 residues). The following search parameters were used: Iodoacetamide derivative on cysteine, 11-plex tandem mass tag® (TMT) on lysine were set as fixed modifications, deamidation on asparagine and glutamine, oxidation on methionine, phosphorylation on serine, threonine and tyrosine as well as Carbamylation and 11-plex TMT on peptide-N-term were set as variable modifications. Monoisotopic masses were searched within unrestricted protein masses for tryptic enzymatic specificity. The peptide mass tolerance was set to ±5 ppm and the fragment mass tolerance to ±15 ppm. The maximal number of missed cleavages was set to 2. The result was filtered to 1% FDR on protein level using Percolator algorithm integrated in Thermo Proteome Discoverer. The localization of the modification sites within the peptides was performed with the tool ptmRS, which is based on phosphoRS (18). Peptides were quantified based on Reporter Ion intensities extracted by the "Reporter Ions Quantifier"-node implemented in Proteome Discoverer. Proteins were quantified by summing unique peptides.

To distinguish regulated phospho-peptides that are altered due to regulation of the phosphorylation site from changes due to regulation of the underlying protein, PSMs were separated into those acquired from phospho-enriched samples and others acquired from the input before enrichment to capture changes on the proteome level. Therefore, proteins were quantified by summing unique and razor peptides detected and quantified in the input fractions. Resulting values were sum normalized across samples. Fractions of the phospho-enrichment were grouped and summarized on the peptide isoform level.

Subsequently, phosphopeptide regulations determined in the enriched fractions were corrected by regulation of the corresponding protein in the input fractions to correct for

changes observed due to regulation on the proteome level. This correction was only applied if at least 3 spectra for the corresponding protein were identified. Statistical significance of differentially expressed phosphopeptides was determined using LIMMA (14) and FDR curves determined in Hein *et al* (1) were applied to extract significantly regulated phosphopeptides. MS/MS spectra assigned to the Tau peptide phosphorylated on residue S214 were validated manually. Most of them showed a mix of different isoforms, mainly of phosphorylated T212 and S214, while some spectra did not contain fragments that allowed for the unambiguous distinction between the possible isoforms.

Data availability

The phosphoproteomics data have been deposited to the ProteomeXchange Consortium via the PRIDE (16) partner repository with the data set identifier PXD065759.

Glutaraldehyde crosslinking

Purified MAST1^{DK,} MAST1^{D} p-S90, 14-3-3η, and co-purified MAST1^{DK}:14-3-3η complex was incubated either alone or in combination, at a final concentration of 20 μM, with 0.01% glutaraldehyde in buffer (50 mM HEPES pH 7.4, 150 mM NaCl, 1 mM TCEP), at room temperature for up to 30 min. The reaction was quenched with 20 mM Tris pH 7.5.

Generation of MAST1 G519S mouse model

Animals used in this study were housed at the IMP/IMBA animal facility under a 12:12 light-dark cycle. Food was provided ad-libitum. G519S MAST1 transgenic mice were generated by CRISPR/Cas9 editing through pronuclear microinjection using Cas9 mRNA, sgRNA, and a single-stranded DNA repair template (19). See supplementary table 1 for gRNA and repair template sequences. The gRNA target sequence was cloned into PX458 plasmid as described previously (20) and sgRNA was expressed using the MEGAshortscript™ T7 Transcription kit (Thermo Fisher Scientific, AM1354). The ssDNA repair template was ordered from Integrated DNA Technologies (Alt-R™ HDR Donor Oligo). Injections were performed on BL6/CBA F1 zygotes. Successfully edited mice were identified using Sanger sequencing and backcrossed using C57BL/6J mice for at

least 10 generations. All procedures were carried out in accordance with the legal requirements under an approved license (M58/006093/2011/14).

Solubility analysis of MAST1 mutants

MAST1^{D} ΔS90WT, E194del, K276del, and L278del were transformed into BL21 Star *E. coli* and grown at 37°C to an OD of 0.7, before induction with 100 µM IPTG and overnight expression at 18°C. Cell pellets from 10 mL of expression culture were lysed in 2 mL of lysis buffer (50 mM HEPES pH 7.4, 150 mM NaCl, 1 mM TCEP, 2 mM benzamidine, 2 mM $MgCl_2$, 1x protease inhibitor cocktail (Sigma P8849), 0.2 kU Denarase, 2 mg lysozyme). Lysates were sonicated three times for 30 sec at 20% amplitude. A sample was taken for SDS-PAGE (whole cell lysate). The lysate was centrifuged at 38,397 g, 4°C, for 15 min, and a sample of the cleared lysate was taken for SDS-PAGE (supernatant). The pellet was resuspended in the same volume of buffer (50 mM HEPES pH 7.4, 150 mM NaCl, 1 mM TCEP, 0.25% CHAPS) by vigorously vortexing, and a sample was taken for SDS-PAGE (pellet).

Western blot

Mouse P0 cortical lysates were prepared using lysis buffer (20 mM Tris pH 7.5, 100 mM NaCl, 10% Glycerol, 1% Triton-X100) and 1:100 Halt™ Protease & Phosphatase Inhibitor Cocktail (Thermo Fisher Scientific, #78444). Brains were lysed by addition of 10 µL lysis buffer per mg of sample, addition of a Tungsten carbide bead and shaking using a TissueLyser II machine (Qiagen; 20 Hz, 2 x 60 s, RT) followed by further incubation on a rotating tube holder (60 min, 4°C). Protein concentrations were determined using a Pierce™ BCA Protein Assay Kit (Thermo Fisher Scientific, #23225). 30 µg of protein lysate was prepared in Laemmli loading buffer + 2% β-Mercaptoethanol and boiled for 5 min at 95°C. Gel electrophoresis was carried out using Novex™ WedgeWell™ 8-16% Tris-Glycine gel (Thermo Fisher Scientific, # XP08160BOX) and Tris-Glycine SDS running buffer (25 mM Tris, 240 mM glycine, 0.1% SDS, pH 8.3) for 2 h at 120 V. Blotting was done on activated PVDF membranes via wet transfer in transfer buffer (31 mM Tris, 240 mM Glycine, 0.025% SDS, 20% Methanol) for 16 h at 20 V. Membranes were blocked used 5% skimmed milk in TBS-T for 1 h @ RT. Incubation with primary antibody was performed overnight, 4°C in blocking solution. The following primary antibodies were used: Anti-MAST1 (ProteinTech, 13305-1-AP, 1:1000), Anti-MAST2 (SantaCruz, sc-377198, 1:300), Anti-MAST3 (Novus Biologicals, NBP1-82993, 1:500), Anti-GAPDH

(Millipore, MAB374, 1:2000). Membranes were washed with TBST and incubated for 1 h, RT with the following secondary antibodies: Goat anti-mouse HRP (Abcam, ab6823, 1:5000), Goat anti-rabbit HRP (Abcam, ab6721, 1:10000). Chemiluminescent imaging was carried out using the ECL™ Western blotting reagent (Sigma Aldrich, # GERPN2109) on a Chemidoc-MP Bioimaging system (Bio-Rad). Quantification was performed using ImageJ.

Supplementary References

1. M. Y. Hein, *et al.*, A human interactome in three quantitative dimensions organized by stoichiometries and abundances. *Cell* **163**, 712–723 (2015).

2. G. La Manno, *et al.*, Molecular architecture of the developing mouse brain. *Nature* **596**, 92–96 (2021).

3. E. Krötenheerdt, *et al.*, Mechanism of activation of an ancestral TEC kinase by PIP $_3$. *bioRxiv* (2025).

4. R. L. Dunbrack Jr, Rēs ipSAE loquunt: What's wrong with AlphaFold's ipTM score and how to fix it. *bioRxivorg* (2025).

5. X. Yang, *et al.*, Structural basis for protein-protein interactions in the 14-3-3 protein family. *Proc. Natl. Acad. Sci. U. S. A.* **103**, 17237–17242 (2006).

6. J. L. Johnson, *et al.*, An atlas of substrate specificities for the human serine/threonine kinome. *Nature* **613**, 759–766 (2023).

7. J. Yang, *et al.*, Crystal structure of an activated Akt/Protein Kinase B ternary complex with GSK3-peptide and AMP-PNP. *Nat. Struct. Biol.* **9**, 940–944 (2002).

8. M. A. Lancaster, *et al.*, Guided self-organization and cortical plate formation in human brain organoids. *Nat. Biotechnol.* **35**, 659–666 (2017).

9. J. Rappsilber, M. Mann, Y. Ishihama, Protocol for micro-purification, enrichment, pre-fractionation and storage of peptides for proteomics using StageTips. *Nat. Protoc.* **2**, 1896–1906 (2007).

10. A. T. Kong, F. V. Leprevost, D. M. Avtonomov, D. Mellacheruvu, A. I. Nesvizhskii, MSFragger: ultrafast and comprehensive peptide identification in mass spectrometry-based proteomics. *Nat. Methods* **14**, 513–520 (2017).

11. F. Yu, S. E. Haynes, A. I. Nesvizhskii, IonQuant enables accurate and sensitive label-free quantification with FDR-controlled match-between-runs. *Mol. Cell. Proteomics* **20**, 100077 (2021).

12. F. da Veiga Leprevost, *et al.*, Philosopher: a versatile toolkit for shotgun proteomics data analysis. *Nat. Methods* **17**, 869–870 (2020).

13. D. Hollenstein, M. Hartl, MsReport. [Preprint] (2025). Available at: https://zenodo.org/records/15309091.

14. M. E. Ritchie, *et al.*, limma powers differential expression analyses for RNA-sequencing and microarray studies. *Nucleic Acids Res.* **43**, e47 (2015).

15. D. Hollenstein, M. Hartl, XlsxReport. [Preprint] (2025). Available at: https://zenodo.org/records/15129819.

16. Y. Perez-Riverol, *et al.*, The PRIDE database resources in 2022: a hub for mass spectrometry-based proteomics evidences. *Nucleic Acids Res.* **50**, D543–D552 (2022).

17. V. Dorfer, *et al.*, MS Amanda, a universal identification algorithm optimized for high accuracy tandem mass spectra. *J. Proteome Res.* **13**, 3679–3684 (2014).

18. T. Taus, *et al.*, Universal and confident phosphorylation site localization using phosphoRS. *J. Proteome Res.* **10**, 5354–5362 (2011).

19. H. Wang, *et al.*, One-step generation of mice carrying mutations in multiple genes by CRISPR/Cas-mediated genome engineering. *Cell* **153**, 910–918 (2013).

20. F. A. Ran, *et al.*, Genome engineering using the CRISPR-Cas9 system. *Nat. Protoc.* **8**, 2281–2308 (2013).
